# Gender-based discrimination towards cisgender women in low- and middle-income countries: a scoping review of definitions and measures used in the health literature

**DOI:** 10.1136/bmjph-2024-002276

**Published:** 2026-06-30

**Authors:** Laurène Petitfour, Swati Srivastava, Rupal Shah-Rohlfs, Wilm Quentin, Manuela De Allegri

**Affiliations:** 1 Heidelberg Institute for Global Health, University Hospital and Medical Faculty, Heidelberg University, Heidelberg, Germany; 2 Inserm, IRD, SESSTIM, Sciences Economiques &; Sociales de la Santé &; Traitement de l’Information Médicale, ISSPAM, Aix-Marseille Universite, Marseille, France; 3 Chair of Planetary and Public Health, University of Bayreuth Faculty of Law Business & Economics, Bayreuth, Germany; 4 German West-African Centre for Global Health and Pandemic Prevention (G-WAC), Kwame Nkrumah University of Science and Technology, Kumasi, Ghana

**Keywords:** Public Health, Sex Factors, Scoping Review

## Abstract

**Introduction:**

Gender-based discrimination (GBD) has pervasive effects on women’s health outcomes. Measurement of GBD is essential in order both to understand and monitor progress against GBD and to study its relationship with health. We aimed to provide an overview of definitions and measures of GBD used in the literature on health in low- and middle-income countries (LMICs).

**Methods:**

We conducted a mixed-method scoping review following Arksey and O’Malley’s methodological framework. We searched PubMed, Web of Science, CINAHL and EconLit and included studies from 1985 to 2025 if they had an explicit focus on GBD and health, were based in LMICs and if the measure of GBD was described. We excluded reviews and studies focusing on specific health conditions or subpopulations. We extracted information on study characteristics, definitions and measures of GBD (their content and their operationalisation). We synthesised data by identifying groups of similar GBD themes and summarising definitions and measures of GBD.

**Results:**

We included 228 studies. Most studies (n=200) were quantitative or included a quantitative element (n=8, mixed-methods studies). Only 20 studies were qualitative. We identified 11 themes around GBD, namely healthcare behaviours, health state, son preference at birth, disrespect and abuse during childbirth, self-declared discrimination, cultural and legal practices, gender roles, access to economic resources, decision-making, education and violence against women. Most studies (n=148) used only an individual indicator measure to operationalise GBD, while 65 studies used one or more composite indicators. Only 30 studies provided a definition of GBD and these varied considerably.

**Conclusions:**

This is the first review providing an overview of how GBD has been conceptualised and measured in LMICs in the literature on health. Results have several implications: (1) future studies should clearly define GBD when aiming to measure it; (2) a consensus should be reached about what GBD encompasses and (3) a comprehensive measurement tool is needed to capture discrimination with regard to the multiple manifestations of GBD.

**WHAT IS ALREADY KNOWN ON THIS TOPIC**

**WHAT THIS STUDY ADDS**

- Empirical studies on GBD in the health literature are mostly quantitative, and mostly originating from Asia (especially India).
- Few studies provide an explicit definition of GBD. Measures of GBD are generally not comprehensive and mostly capture only one or a few themes regarding GBD (eg, health behaviours, health state).

**HOW THIS STUDY MIGHT AFFECT RESEARCH, PRACTICE OR POLICY**

- In order to accurately measure GBD, researchers need to be explicit in the way they conceptualise it.
- Advancing a standardised conceptualisation of GBD and its underlying domains is essential to improve measurement accuracy and comparability across contexts.

## Introduction

 Gender-based discrimination (GBD) has pervasive effects on women’s health outcomes by shaping power dynamics within and outside the household, and thereby leading to unequal access to essential resources, such as food, education and health services.^1 2^ Emerging evidence suggests that the experience of GBD may contribute to the high levels of maternal mortality still observed in many regions of the world.^3 4^ In 2020, 240 000 women died from preventable causes related to pregnancy and childbirth, and 95% of these deaths occurred in low- and middle-income countries (LMICs) (World Health Organization (WHO)). Gender inequality contributes to maternal undernutrition and unmet needs for healthcare among women, involving harmful social norms and lack of health system responsiveness, often perpetuating gaps in health outcomes.^5^ In several LMICs, studies have revealed that boys benefit more from extended periods of breastfeeding than their sisters, leading to better health outcomes.^6 7^ Gender inequity is present from the moment of birth; as in several countries, sex ratios have been reported to deviate already at birth where we would expect a biological ratio of approximately 105 boys to 100 girls and instead we observe a divergence ranging from 2% to 8% of this value.^8^

GBD can be defined as ‘any situation where a person is denied an opportunity or misjudged solely on the basis of their sex and/or gender identity’.^9^ GBD can be direct, for example, when a difference in treatment is based explicitly on gender; or indirect, which occurs when a law, policy, programme or practice appears to be neutral but has discriminatory effects when implemented.^10^ Reducing GBD sits at the core of goal 5 (gender equality) and goal 10 (reduced inequalities) of the Sustainable Development Goals (SDGs). In addition, reducing GBD against women is postulated as an essential step towards achieving Target 3.1 of the SDGs, which demands a strong decrease in maternal mortality by 2030.^11^ Given that both gender and health intersect across multiple SDGs, enhancing health equity and reducing maternal mortality relies on reducing GBD; and evidence suggests that greater gender equality has a mostly positive effect on the health of females.^12^

To date, no consensus has been reached in the health literature on the definition of GBD, on what it encompasses and on how it can be measured in a comprehensive way. Yet, measurement of GBD is essential to understand and monitor progress against it and to determine its effects on health. International actors appear to have different conceptions of GBD, some being legal,^13^ focused on gender equality^14^ or on social norms.^15^ In addition, a number of macro-level tools and indices have been created to assess GBD like the Gender Development Index (GDI) and the Gender Inequality Index (GII), both developed by the United Nations Development Program (UNDP), the Social Institutions and Gender Index (SIGI) developed by the Organization for Economic Cooperation and Development (OECD) and the Global Gender Gap Index developed by the World Economic Forum,^16^,^18^ which allow international comparisons and tracking of temporal changes. However, they do not capture the diversity of channels through which GBD affects individual health outcomes, due to the limited numbers of health-related elements they include.

To our knowledge, there is no available overview of either GBD measures or the specific dimensions and aspects of GBD in the context of LMICs. The only available review on the topic has focused on high-income settings and the influence of GBD on mental health outcomes.^19^

Given this backdrop, we conducted a comprehensive scoping review to elucidate the definitions and measures of GBD used in the health literature specifically targeting LMICs. In order to ensure the coherence of our review, and considering also our human resources, we chose to focus on discrimination against cisgender women. Given the intersectionality between gender-based and other forms of discrimination, we focused on cisgender women and excluded discrimination against transgender individuals. Discrimination against transgender individuals represents a growing field of literature and is increasingly recognised as a standalone area of research, due to the specificity of the stigma and experiences it encompasses.^20 21^

## Methods

### Research questions and selection of the studies

We followed Arksey and O’Malley’s six-step methodological framework for designing and conducting the scoping review,^22 23^ and reported our review following the Preferred Reporting Items for Systematic Reviews and Meta-analyses extension for Scoping Reviews guidelines.^24^ The protocol of our scoping review has been registered on Open Science Framework (Registration Number: osf.io/t97f8) and previously published.^25^

We defined the specific research questions (step 1 of Arksey and O’Malley’s framework) as:

1. How is GBD conceptualised, described, and defined in the health literature? Which specific aspects and components of GBD are considered?
2. In quantitative studies, how is GBD measured (eg, dimensions considered, data sources and data collection modalities, analytical approach)?
3. In qualitative studies, how is GBD defined? Which dimensions and concepts of it are explored, why and how?
4. What measures and what dimensions are considered in specific settings and/or for specific health outcomes?

We searched four databases in October 2021, including PubMed, Web of Science, CINAHL and EconLit (step 2 of Arksey and O’Malley’s framework). Search terms were related to ‘gender discrimination’, ‘health’ and ‘developing country’. Search terms are provided in online supplemental annex 1. We updated the search in July 2025 following the same protocol. We considered only peer-reviewed articles published in English between 1 January 1985 and 19 July 2025. We included all measures of GBD used in the included studies, not only measures of GBD that would relate to health. Nevertheless, we included only studies mentioning health as a focus, as we were interested in studies that had examined GBD in relation to health status and/or access to healthcare services.

Four reviewers independently screened titles and abstracts for study eligibility. A full-text review was then conducted by the same four reviewers. At each stage, two reviewers were required to approve an article in order to progress to the next step, and conflicts were discussed as a group and resolved either by consensus or by adjudication by a fifth reviewer. Studies were included if they focused on GBD as a variable either of interest or of exposure, were based in LMICs and had an explicit focus on discrimination (step 3 of Arksey and O’Malley’s framework). We used the World Bank classification to define LMICs.^26^ We excluded studies where discrimination was caused by factors other than gender (socioeconomic factors, subpopulation, specific health conditions). In these cases, the measure being used captured the intersectionality between gender and a specific health condition or for a specific group (for instance, diabetic cisgender women or elderly women). Due to the difficulty to disentangle the portion of GBD attributable solely to gender versus other factors, we limited our review to studies addressing cisgender women in general. This approach allowed us to maintain a clearer focus on GBD as defined by our research question. We also excluded studies where GBD was not a main focus, so that the GBD measure for quantitative measures or concept for qualitative ones would be detailed enough for data extraction. We described all criteria in a published protocol,^25^ and there was no deviation from this protocol. All screening was conducted in Covidence.^27^ We also searched reference lists of included studies and excluded systematic reviews.

### Extraction and analysis of the data from the selected studies

We designed a data extraction table (step 4 of Arksey and O’Malley’s framework) with two main parts. The first part gathered general information about the study: the setting (geographical and time period), study type (quantitative, qualitative or mixed-method), objectives, outcome and exposure variables, the main results, and, if any, the conceptual framework used, and the definition of GBD used.

The second part gathered detailed information about the GBD measures used by the study. For quantitative studies, we extracted detailed information about how data was collected (survey data, associated questionnaire items and/or secondary data and its sources), how the GBD variable was computed and the method(s) with which it was analysed. For qualitative studies, we extracted information about data collection (focus group discussion, individual interviews, etc), the elements of the study tools related to GBD, the way GBD was conceived and the thematic and analytic approaches.

To chart the data (step 5 of Arksey and O’Malley’s framework), we separated the included studies into groups of similar studies. First, we distinguished quantitative from qualitative studies (mixed-method studies being included in each group, with quantitative and qualitative components analysed separately). Then, we demarcated two subgroups of quantitative studies: one where GBD was operationalised by a single variable or several separate variables (‘individual indicator studies’), and the second where GBD was operationalised through a composite index summarising several items (‘composite index studies’).

We then listed separately: (1) for the individual indicator studies, every indicator used; (2) for the composite index studies, every item included in the GBD indices and (3) for the qualitative studies, all the elements of the GBD concept. From these three lists, two reviewers separately gathered items into homogenous thematic groups and defined a title for each group based on the terminology used by the included studies. Subsequently, agreement on thematic titles was reached within the research team through several rounds of discussions and revisions, leading to a final comprehensive list of GBD themes covered in the included studies.

### Patient and public involvement in research

No patients or public were involved in this study.

## Results

We present our results as follows. First, we provide a global overview of the included studies (type of study, geographical setting, date of publication and methodological approach) and of the GBD themes we found in the included articles. Second, we describe the measures of GBD in more detail.

For each group of studies, we describe: (1) how GBD is conceptualised and defined and (2) how these concepts are operationalised and implemented.

Last, we provide an overview of GBD measures used in the context of different settings and health issues.

### Number of articles included in each step of the review

**Figure 1:**
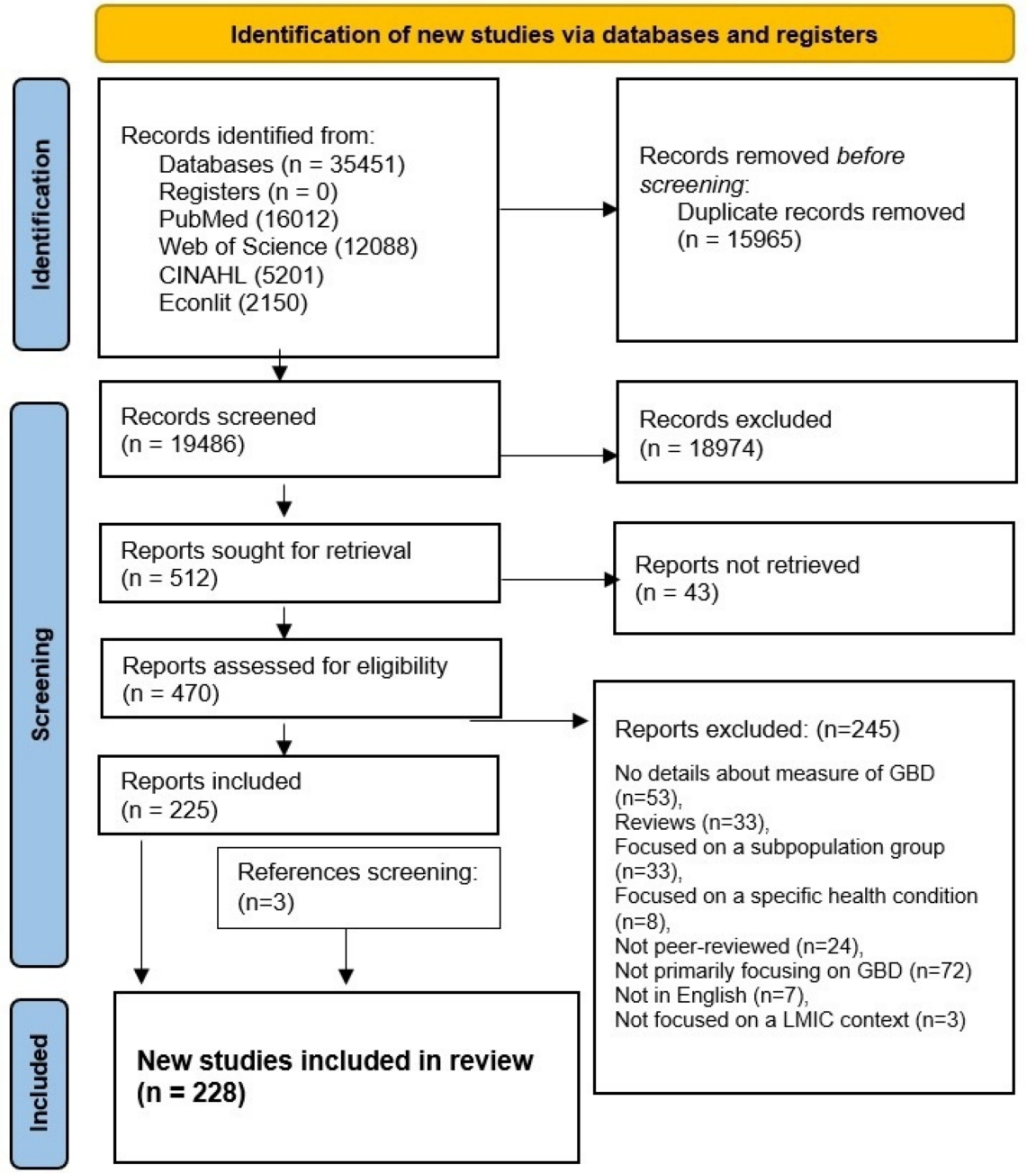
PRISMA chart of the scoping review. GBD, gender-based discrimination; LMICs, low- and middle-income countries; PRISMA, Preferred Reporting Items for Systematic Reviews and Meta-Analyses.

The search identified a total of 35 451 titles in the different databases. After a removal of duplicates, 19 486 titles and abstracts were screened, of which 18 974 were excluded because they did not focus on GBD or were from high-income countries. We then sought to retrieve 512 full texts but were unable to obtain reports of 43 studies. The remaining 470 full texts were screened for eligibility, and 245 studies were excluded because they did not provide details about their measure of GBD (n=53), were reviews (n=33), focused on a subpopulation group (n=33), were on a specific health condition (n=8), were not peer-reviewed publications (n=24), were not primarily focusing on GBD (n=72) or were not in English (n=7). After the inclusion of three additional papers identified through reference screening, we retained 228 articles for data extraction and analysis (figure 1).

### Overview of included studies

**Figure 2:**
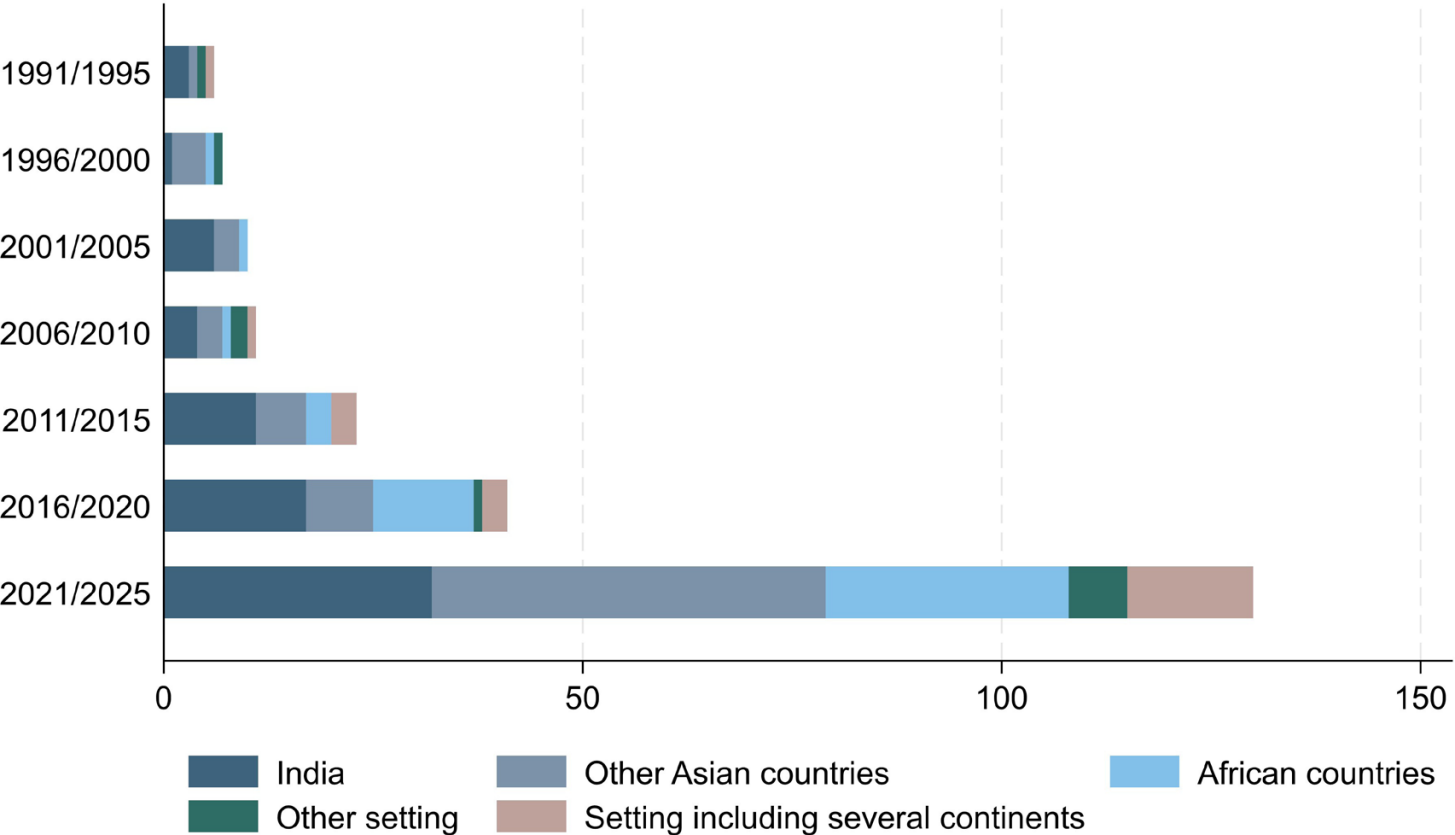
Distribution of geographic setting and year of publication of included studies.

The full list of studies and extracted information is included in online supplemental annex 2. The scientific literature linking GBD to health outcomes has grown considerably since 1991 (first included study) (figure 2). From the 228 original studies included in our review, three-quarters (n=194) were published after 2010.

Most studies (200 studies or 88%) were quantitative,^26 7 28^,^195^,^196^ ^197^,^198^,^199^,^200^ ^201^,^202^,^203^ ^204^ ^205^ ^206^ ^207^ ^208^,^209^ ^210^,^211^,^212^,^213^,^214^ ^215^,^216^ ^217^,^218^ ^219^ ^220^ ^221^ while only 20 studies (9%) were qualitative^222^,^241^,^242^ and 8 (3%) were mixed-methods.^243^,^250^ Within the group of 208 quantitative or mixed-method studies, 65 studies (29%) used one or more composite measure of GBD, while 143 studies (70%) used a simple individual indicator measure of GBD.

Of the 228 studies reviewed, 72 (64%) focused on Asia, with India being the subject of 74 studies (32%), as shown in figure 2. This trend changed over time: until 2020, India alone was the setting of at least one-third of included studies. From 2021 to 2025, it represented less than one quarter, while the proportion of other Asian settings grew to 36%, as well as the share of African settings which grew from 10% of included studies from before 2016 to 25% of included studies from after 2016. While our primary emphasis was on LMICs, three of the studies also included high-income countries (HICs) owing to their simultaneous research settings in both HICs and LMICs.

Most studies did not offer an explicit definition of GBD. Only 30 studies (13%) provided a definition of GBD, and notably from 2021 to 2025 only six did. All definitions are provided in online supplemental annex 3; the definitions varied considerably. Some studies provided conceptual definitions, anchored in social norms, while some others only gave examples of its manifestations. Some studies did not define GBD in general but specific forms, for example, gender-based violence, disrespect and abuse during childbirth or son preference.

### The GBD themes

Table 1 provides an overview of the GBD themes that emerged across studies included in the review, along with the definition of each theme, and the type of study in which they were used. Online supplemental annex 2 indicates to which theme each included study belonged to. The first theme related to healthcare behaviours, including nutrition, breastfeeding, immunisation, healthcare seeking (occurrence and delay in response of symptoms), healthcare utilisation, healthcare-related expenditures and maternal health (eg, number of antenatal visits).

**Table 1:** List of the themes of gender-based discrimination identified in the included studies

Themes	Definition and example	Used in			
		Quantitative studies with		Qualitative studies	Total
		Individual indicator	Composite index		
1. Healthcare behaviour	Any form of health-related behaviour, including healthcare seeking and utilisation, breastfeeding, nutrition, healthcare financing strategies, maternal health, immunisation Example: girls and women have poorer access to nutrition (including breastfeeding when infant), immunisations and appropriate healthcare seeking when sick	49 (47 quantitative, 2 mixed-method)	1 (1 quantitative)	3 (2 qualitative, 1 mixed-method)	53
2. Health state	Indicators that reflect the health state: morbidity (including occurrence of symptoms and assessing stunting through anthropometric measures) and mortality Example: girls and women exhibit poorer health state, poorer anthropometric indicators than boys and men	54 (52 quantitative, 2 mixed-method)	1 (1 quantitative)		55
3. Son preference at birth	Measures son preference at birth: individual stated preferences or population ratios Example: due to selective abortion and differential antenatal care and contraception strategies, the proportion of boys and girls at birth and during the first month of life is imbalanced compared with a hypothetical situation without GBD	59 (47 quantitative, 2 mixed-method)		3 (2 qualitative, 1 mixed-method)	62
4. Self-declared discrimination	Declaration of experienced GBD Example: women declare that they were discriminated due to their gender	1 quantitative			1
5. Cultural and legal practices	Explicit (legal) and implicit (tradition, social norms) practices Example: women have less rights or more duties than men, which affects their ability to care for their health as they would need to	2 quantitative	7 (6 quantitative, 1 mixed-method)		13
6. Gender roles	Perceptions of feminine and masculine characteristics, responsibilities of each gender within or outside the household Example: gendered attitudes regarding female and male nature and prerogatives that shape harmful behaviours towards health.	1 quantitative	12 (11 quantitative, 1 mixed-method)	9 (7 qualitative, 2 mixed-method)	22
7. Access to economic resources	Women’s access to property, land, inheritance and to the labour market Example: women have lower financial resources and autonomy, which affects their nutrition and health behaviours, and ultimately their health.	4 quantitative	12 quantitative		16
8. Decision-making	Bargaining power of women within their household, as well as the capacity of women to make decisions about their own life. Example: women must get the authorisation from their husband or another person in various aspects of their life, including healthcare issues, depriving them of their agency.	2 quantitative	22 (11 quantitative, 1 mixed-method)	2 qualitative	24
9. Education	Women’s educational level and exposure to media Example: by being less educated, women have access to less or poorer information, and can be disrespected, including by healthcare providers.	4 quantitative	5 quantitative		9
10. Violence against women	Mistreatment of women, including verbal, physical or sexual abuse. Example: domestic violence and tolerance regarding violence against women, under its various forms, is an extreme manifestation of GBD	7 quantitative	39 (37 quantitative, 2 mixed-method)	5 (4 qualitative, 1 mixed-method)	50
11. Disrespect and abuse during childbirth	Experiences of women during childbirth, provider attitudes towards abuse during childbirth Example: disrespect and abuse during childbirth is the manifestation of a lower standard of care a specialty with an exclusively female audience		14 (11 quantitative, 3 mixed-methods)	11 (8 qualitative, 3 mixed-method)	25
Total*		148*	65	28	236†

* In each column, the numbers do not add up to the total because studies can tackle several themes.

† The total is 236 and not 228 studies, because we separated the two analyses from mixed-method studies (n=8) for clarity.

GBD, gender-based discrimination.

The second theme considered health state measures used as proxies for GBD, including the occurrence of symptoms, anthropometric indicators of long-term malnutrition or stunting and mortality indicators.

The third theme captured son preference at birth and included indicators reflecting parental preference for sons over daughters (eg, a higher reported ideal number of sons than daughters), strategies aimed at having a son (eg, birth spacing through contraception or breastfeeding duration, differential antenatal care) and sex imbalances at birth. Some studies used these indicators to estimate the number of ‘missing women’, referring to girls who were never born.

The fourth theme included a single study in which discrimination was self-reported by respondents.

The fifth theme related to cultural and legal practices. Cultural practices included norms privileging men within the household (eg, acceptance of polygyny or endorsement of sexist statements). Legal practices referred to indicators capturing the existence of laws protecting women’s rights and autonomy (eg, rights to divorce, child custody, abortion, protection against violence and political representation, such as quotas for women in parliament).

The sixth theme concerned gender roles, including attitudes and perceptions regarding socially constructed or normative characteristics attributed to men and women (eg, agreement with statements such as ‘Men should be career-oriented, whereas women should be family-oriented’).

The seventh theme focused on women’s access to economic resources, including legal rights to property, inheritance, or credit, as well as self-reported ownership of land or bank accounts and employment status.

The eighth theme captured women’s decision-making power within the household, particularly regarding healthcare, reproductive health and the use and allocation of household resources.

The ninth theme related to educational attainment as an indicator of GBD.

The tenth theme concerned violence against women, including both attitudes tolerating violence and self-reported experiences of physical, sexual or verbal abuse.

The eleventh theme addressed disrespect and abuse during childbirth. These indicators were derived from observations in healthcare facilities, women’s self-reports and healthcare workers’ reported attitudes regarding substandard care during childbirth.

Healthcare behaviours, health state, son preference at birth and violence against women were the most frequently represented themes (with 53, 55, 62 and 60 studies, respectively, for themes 1, 2, 3 and 10). However, we observed substantial variations across regions and over time.

Healthcare behaviour and health state indicators each accounted for nearly half of the literature on GBD and health during the first 25 years of the study period. From 2011 onwards, the proportion of studies focusing on health status declined to below 20%, and from 2016, the share focusing on healthcare behaviours also fell below 20%. Approximately 30% of studies conducted in Asian settings addressed healthcare behaviours and a similar proportion addressed health state, whereas these proportions were lower in African settings (10%). Additionally, 33% of multicountry studies measured GBD using health state indicators and conducted cross-country comparisons. The theme of son preference at birth was present throughout the study period and was particularly prominent in studies published between 2021 and 2025 (approximately one-third). These studies were predominantly conducted in Asian settings, especially in India. However, between 2016 and 2025, such measures were increasingly used in other Asian countries, including China, Bangladesh and Nepal.

Other GBD themes emerged more prominently after 2011. Decision-making indicators were used in 12% of studies published between 2016 and 2025; violence against women indicators in 31% of studies published between 2021 and 2025 and disrespect and abuse during childbirth indicators in 33% of studies published between 2016 and 2021. Geographic patterns also varied: indicators related to access to economic resources were more frequently used in multicountry studies than in single-country studies; decision-making indicators were primarily used in Asian contexts (excluding India) and in African settings; violence against women was studied across all regions and disrespect and abuse during childbirth were more commonly examined in African contexts.

In the later sections of the manuscript, we review how single or multiple indicators were compiled to generate single indicators or composite indices. We compiled this list exclusively on the basis of the data extracted from the 228 articles included in the review, by grouping items found in the single survey/tools against some higher-level themes, to allow a simple broader categorisation of the corpus of literature under analysis. Some themes were found in two groups of studies. For instance, disrespect and abuse during childbirth was tackled both through composite quantitative indices and qualitative studies. Similarly, violence against women and gender roles were present both in composite indices and in qualitative studies. Also, healthcare behaviour or son preferences were measured by quantitative individual indicator studies and also explored in qualitative studies.

Conversely, certain themes were only found in either qualitative or quantitative studies, but not in both. This includes areas such as access to economic resources, education, as well as cultural and legal practices, which were consistently examined as components of composite indices. The sex ratio at birth, widely used as an individual indicator GBD measure, was introduced in one country-level index (the Women, Peace and Security Index (WPSI)).^48^ Variables related to gender-differences in health measures, such as immunisation rates or anthropometric measures, were introduced as individual indicators in the analytical approach in some studies but were not included in composite indices.

### Quantitative studies

#### Individual indicator measures

##### Conceptualisation

Online supplemental table 1 presents a summary of the indicators used by the 148 studies that relied on individual indicators to measure GBD. We list all measures that made use of a single indicator to capture GBD, whether these measures were implemented to reflect an exposure variable, an outcome variable or to explain the distribution in the population along a given gradient. For instance, differences in breastfeeding practices between male and female infants could be taken as exposure to measure later cognitive and physical development; or could be taken as an outcome variable to measure exposure to cultural practices; or could simply be documented. These indicators are categorised into three different operationalisation approaches described below.

##### Operationalisation and implementation

We distinguished three ways of operationalising the measure of GBD among the 148 individual indicator studies. 49 studies (33%) estimated the impact of gender on an outcome by introducing a gender dummy in an econometric regression and interpreting the significance of the estimated coefficient. They later interpreted the significance of this dummy variable and its value as a measure of GBD in a given setting.

In 59 studies (40%), GBD was operationalised as the existence of different values for males and females for a range of different measures, such as immunisation rates, the prevalence of any symptom or mortality. In these studies, GBD was the outcome of the analysis in 50 studies (85%), and the exposure in 9 studies (15%).

Lastly, 40 studies (27%) operationalised GBD as a standalone variable, such as the proportion of women who reported more boys than girls in their ideal family size, or the number of missing women. The variable was the outcome of the analysis in 28 of these studies (70%), and the exposure in 12 (30%).

When studies used more than one indicator (for instance, several vaccination rates to measure immunisation coverage or several anthropometric measures to capture nutritional status), the same estimation approach was replicated for each indicator.

### Quantitative studies with composite indices

#### Conceptualisation

In the 65 studies using composite indices, authors defined and measured GBD using an index that aggregated multiple indicators of different GBD themes into a single variable. Online supplemental table 2 provides an overview of the GBD themes covered by the composite index used in the 65 studies as well as their geographical settings, and the tools that were used.

In five studies, the measurement was performed with countries as the unit of observation.^48 101 132 164 251^ They measured legal and cultural practices using legal rights and national statistics, relying on the existence of laws against GBD to measure it^251^ or on the following indices:

- The GII and GDI from UNDP, which used statistics at the national level, such as maternal mortality ratio, the adolescent birth rate or the share of female seats in Parliament.^48^
- The SIGI from the OECD, which included variables that reflected the existence of laws, such as women’s right to have parental authority, to access property and loans or cultural norms such as acceptance of polygamy or freedom of movement and national statistics, such as the number of missing women or the percentage of women who have undergone Female Genital Mutilation.^101^
- The WPSI from Georgetown Institute for Women, Peace and Security and Peace Research Institute Oslo, which included variables that reflect the existence of laws or cultural norms such as the absence of legal discrimination and national statistics such as the male to female ratio at birth or the perception of community safety.^252^
- The Laws on Violence against women and girls Index, developed to draw international comparisons regarding child marriage, sexual harassment, domestic violence and marital rape.^164^

In the other 60 studies, GBD was computed at the individual level using the following tools.

24 studies used data from the Demographic and Health Survey (DHS) surveys to compute violence against women and decision-making indicators to measure GBD. None of these studies mentioned validation of the computed indicators. Three studies used survey tools with psychological questionnaires validated in various contexts to measure sexist attitudes, namely the Gender Equality Measure (GEM) validated in an Indian setting,^253^ the Ambivalent Sexism Inventory (ASI) validated among US respondents^254^ and the Gender Discrimination Inventory (GDI) validated among Egyptian students.^103^ Conceptually, the GEM tackled gender norms through five aspects (gender, violence, sexuality, masculinities, reproductive health) while the ASI differentiated hostile sexism from benevolent sexism, defined as ‘attitudes towards women that are sexist in terms of viewing women stereotypically and in restricted roles but that are subjectively positive in feeling tone (for the perceiver).’ The GDI adopted a trauma-based approach, considering the consequences of GBD.

A group of studies focused on violence against women: the Violence Against Women Indicator (VAWI) validated among a male population from Sweden,^255^ the revised Conflict Tactics Scale (CTS) validated on student population data from the US^256^ and the Humiliation, Afraid, Rape, Kick (HARK) questionnaire validated in an English context.^257^

Lastly, a group of 14 studies measured disrespect and abuse during childbirth following the landscape analysis introduced by Bowser and Hill,^258^ further developed^259^ and validated in Nigeria, Ghana, Guinea and Myanmar.^42^

The use of a composite measure does not automatically translate into taking several themes of GBD into account. 46 studies (68%) employed a composite measure to measure a single GBD theme, like decision-making, violence against women or disrespect and abuse during childbirth. Only five studies used composite indices to capture five^43 58 260^ or six (the maximum)^245 261^ GBD themes.

#### Operationalisation and implementation

Among the 65 studies that used a composite GBD measure, 44 studies (68%) used the GBD measure as an outcome variable of the analysis. In 17 studies (26%), it was an exposure variable, and the outcome variable was a variable linked to health state or health seeking behaviour. Finally, four studies (6%) used two distinct composite measures of GBD as exposure and outcome variables. Specifically, they study the impact of gender norms on the experience of abuse during childbirth^50 245^ and the experience of violence.^67^

Some tools contained self-declared questions on decision-making habits in the household (eg, in the DHS, ‘In your household, who has the most say on…?’), agreement with tolerant attitudes towards violence against women (eg, in the DHS, ‘Is it justified for a husband to beat his wife if…?’). Tools about gender roles were made of statements with which respondents are asked the extent to which they agree. They might be statements about gendered roles or characteristics (‘A man should have the final word about decisions in his home’ in the GEM, ‘A woman’s role is taking care of her home and family’ in the ASI), perceptions (‘I am disappointed in myself because I am a female’ in the GDI). Other tools, focusing on violence against women directly asked about the occurrence of precise experiences over a certain period of time (eg, ‘My partner insulted me in a way that made me feel bad’ in the VAWI and in similar ways in the HARK and the revised CTS).

The tools following Bohren *et al*^259^ and measuring disrespect and abuse during childbirth included the observation of deliveries and individual surveys administered to women who experienced maternal care.

### Qualitative studies

#### Conceptualisation

We grouped the 28 studies with a qualitative component into five thematic categories. 11 studies (39%) focused on disrespect and abuse during childbirth; 3 studies (11%) investigated women’s access to and use of health services (associated with health behaviours category); 5 studies (18%) looked at how violence against women is experienced and tolerated within communities and societies; 8 studies (29%) examined gender roles and the position of women in the family and in society; and finally, 2 studies (7%) explored preference for sons.

#### Operationalisation and implementation

All 28 studies used focus group discussions and individual interviews as a data collection method. 18 (64% of qualitative) studies were anchored in sub-Saharan African settings and 5 (18%) in India. Other settings include Papua New Guinea, Bangladesh and Sri Lanka.

Most studies focused on women’s experiences and perspectives when dealing with violence against women and disrespect and abuse during childbirth (only three included interviews of healthcare providers), while all studies dealing with gender roles or violence confronted male and female perspectives.

Analytical frameworks were diverse and depended on the specific topic. Studies focusing on disrespect and abuse during childbirth constituted the most homogeneous group, as eight of them referred to Bohren’s framework, meaning that the interview guides included questions corresponding to each component of the framework.

Among studies examining gender roles, three applied the Gender Analysis Framework to explore relationships between women and men, as well as gender roles within families, communities and society in specific contexts and across various domains.^262^ The remaining studies drew on distinct theoretical approaches, including feminist analysis (focusing on gender and power relations), social norms analysis using vignettes and eco-social approaches to violence against women.

### Additional methodological considerations

**Figure 3:**
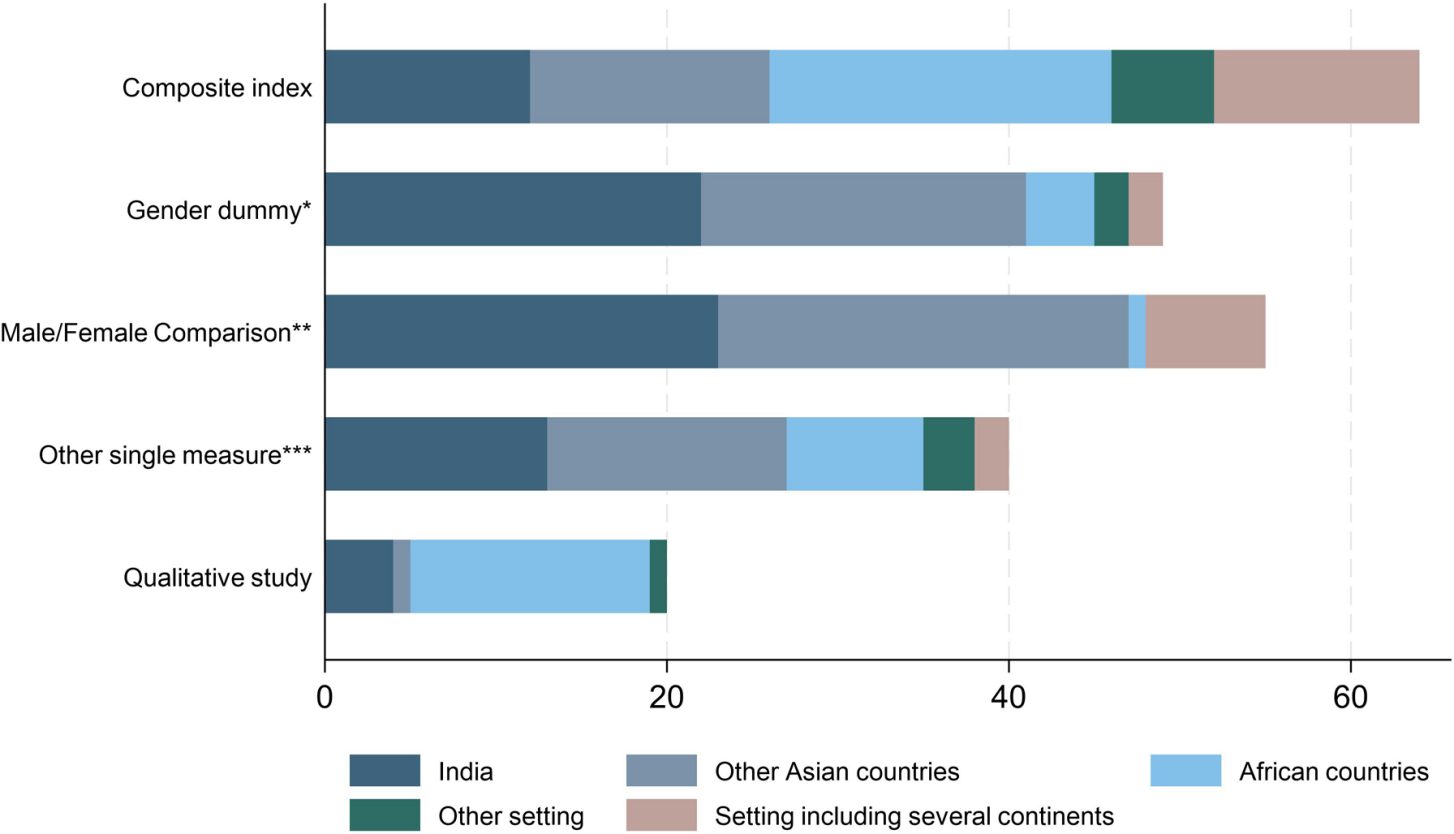
Distribution of geographical setting per type of study.

The representation of geographical settings across study types and measures of GBD revealed distinct patterns in the literature. Asian settings, particularly India, are disproportionately represented in analyses based on individual GBD indicators, especially those using male-female comparisons or including gender as a dummy variable in regression models (figure 3).

In contrast, studies employing composite indices draw on a more diverse set of geographical contexts. African settings, meanwhile, are more prominently represented in qualitative research.

**Figure 4:**
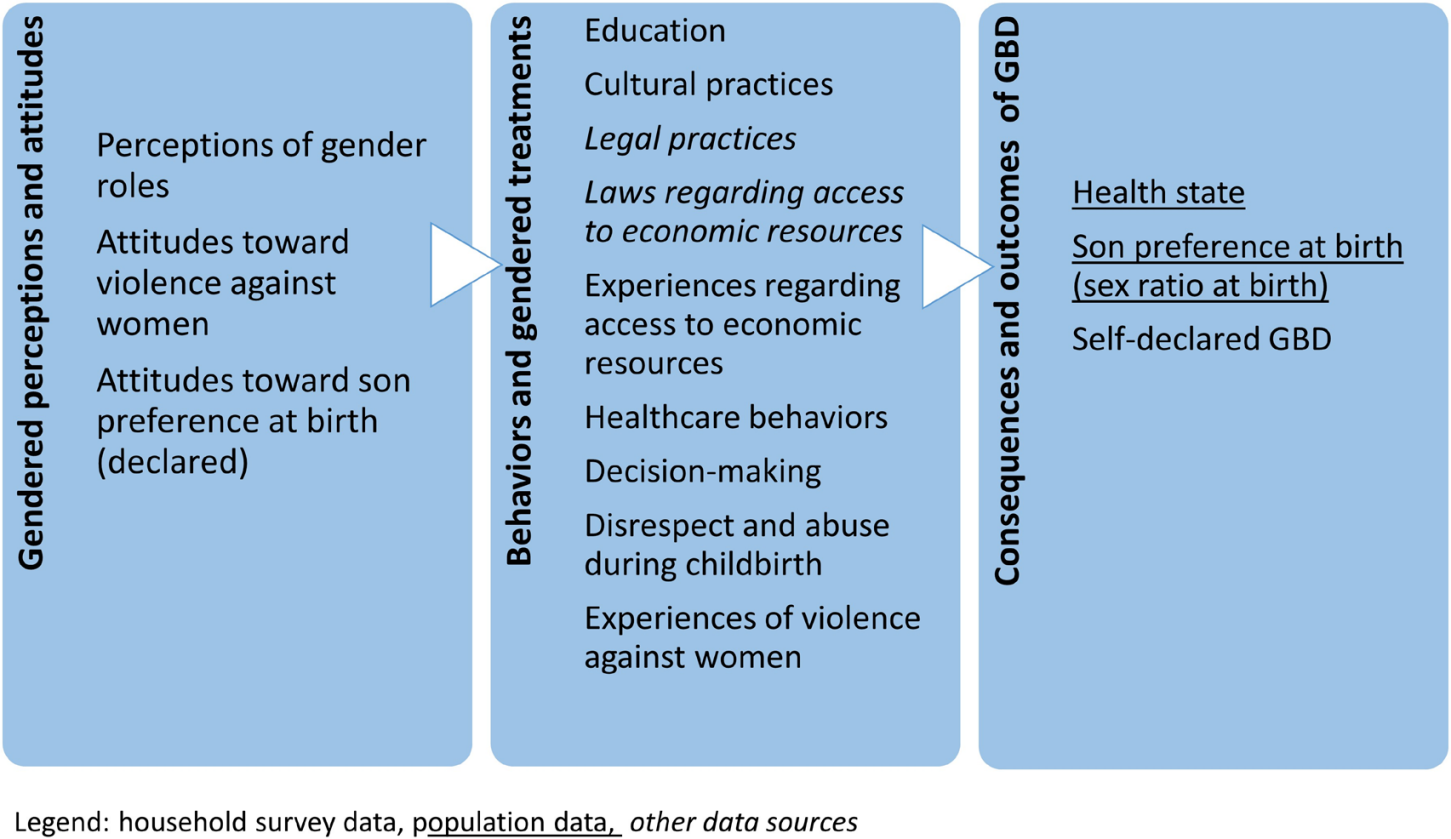
Overview of data sources used for the different themes of gender-based discrimination (GBD).

Second, it also becomes apparent that the use of different measures is associated with the use of different data sources. While attitudes and perceptions of GBD were mostly explored using psycho-social scales, behaviours were assessed using standardised household survey tools (in normal text in figure 4), such as the DHS, and health outcomes were explored using population-based data, such as morbidity and mortality data (underlined text in figure 4).

## Discussion

This study makes a unique contribution by providing the first comprehensive review of how GBD has been conceptualised, defined and measured in the health literature, with specific focus on LMICs. We note that the number of studies increased substantially over time, suggesting increased attention being paid to the relationship between GBD and health. This increase in publications reflects the increased focus that gender equality is receiving outside academia, in the health policy sector, with LMICs increasingly trying to develop strategies to close existing gender inequities in health, and development partners committing to feminist development policies.^263^,^265^ Moreover, we also note that over three quarters of all studies were quantitative in nature, reflecting a desire to quantify the size of the association between GBD and health, and based in Asia, particularly in India. Most studies provided no explicit conceptual definition of GBD, but rather defined GBD via the empirical measure they applied. Quantitative studies often captured GBD by measuring the gender gap in a given health behaviour or outcome. More complex quantitative studies relied on multidimensional indices, computed using household survey data (capturing attitudes and experience regarding GBD) or national level statistics (about health outcomes or legal rights). Qualitative studies generally captured GBD through the prism of disrespect and abuse during childbirth and gender roles, relying on a common set of well-established analytical frameworks. Studies exploring sex preferences were anchored mostly in the Asian setting, especially India and China, while GBD composite indices were distributed evenly across study settings.

Appraising findings from our review against existing evidence, we note a few interesting elements. First, the fact that most of our studies did not include an explicit conceptual definition of GBD resonates with what has been observed before in similar reviews of the global literature.^266^ This absence of a clear and comprehensive conceptualisation of GBD has been pointed out by Shapiro *et al*.^267^ They have criticised the approach whereby a single dummy variable differentiating males and females is used as an exposure variable in a regression model as a proxy of gender bias in medical and epidemiological studies, ignoring the complexity of what this bias really entails. In fact, the absence of a clear definition in most academic studies is surprising given that international institutions and NGOs provide operational definitions of GBD.^13^,^15^

Second, we note that although an explicit definition of what constitutes GBD may be missing in many of the articles we reviewed, the choices made by the authors to operationalise the measurement ultimately revealed the authors’ conceptual standing vis à vis GBD. For instance, although they may appear to be simplistic in their operational implementation, studies that measured GBD by identifying a gender gap in a given behaviour or health outcome implicitly drew from a conceptualisation of GBD inspired from a human capital approach^268^ adopted by Sen to explore the issue of son preference and missing women^269 270^ and summarised by Whitehead *et al*.^271^ Similar to a previous review,^272^ we believe that the literature on son preferences reflects this conceptualisation of GBD.

Third, contrary to the above, measures of GBD that are based on composite indices make an explicit attempt to capture multiple aspects of GBD into a single value. Measures computed at the aggregated (eg, national) level encompass several themes of GBD^273^ but rely on very few indicators for each theme, which constitutes the main limitation to their comprehensiveness. In addition, they fail to capture individual perceptions and experiences. The UNDP, which releases the GII, acknowledged this limitation by enriching their work with the Gender Social Norms Index, relying on data from the World Values Surveys.^274^ Unfortunately, they include only a handful of developing countries, with Sub-Saharan Africa particularly underrepresented. On the opposite, composite measures computed at the individual level allow for a deeper understanding of one aspect or form of GBD but fail to provide a comprehensive measure of GBD. For example, they often focus on one form of GBD (disrespect and abuse during childbirth, violence against women or lack of decision-making) or one approach of GBD (for instance, the psychological one, adopted by the GEM, the ASI and the GDI). Additionally, despite the wide use of the DHS survey data for decision-making and violence against women indicators in Africa and Asia, allowing cross-comparisons across countries, these indicators have not been validated. Only two other composite indicators were validated in LMICs and on general population samples, while perceptions and attitudes appear to be very context specific. This represents a strong limit to their external validity and their capacity to provide reliable comparisons across countries.

Fourth, and last, we note that only a minority of studies employed qualitative and/or mixed-methods methodologies. Although this is not per se surprising and reflects an overall tendency of quantitative research to dominate the health sciences, it is nevertheless a very disconcerting finding. Considering the complexity of the issue at stake, there is no doubt that even in the presence of a single measurement, qualitative research would be fundamental to pilot and adjust tools as well as to contextualise and understand emerging results. The importance of working in a mixed methods context is increasingly being recognised and should be considered in this field of research as well, in order to orient relevant policy options to better address GBD.

The findings presented in our review bear a number of implications for further research and policy. First and foremost, the lack of clarity on how researchers conceptualise GBD and the variety of its experiences and perceptions in women’s everyday life, and the pathways through which they can affect their health emerges as a clear limitation in today’s health literature. It lowers its actionability by leaving its implications open to interpretation in a field where gender proponents in global health organisations constitute a divided crowd, disagreeing on several crucial points.^275^ For instance, some prioritise the reduction of gender inequality while others the fight against harmful gender norms.^276^ Both rely on distinct measures of GBD that reflect their views, partly reflecting the wide range of measures identified by our review. In this context, it is essential that researchers are explicit about the definition of GBD and the concepts they mobilise, so that the evidence they produce helps deliver clear messages.

Second, confronted with the diversity of definitions and measures of GBD, it would be important that a consensus is reached about how GBD is conceptualised and what it encompasses. Reaching such an agreement would represent a fundamental step forward to enable comparability across studies and settings and could represent the first step towards outlining clear pathways through which GBD is thought to produce inequities in health behaviour and health outcomes. Furthermore, agreeing on a definition of GBD could represent the first step towards outlining clear pathways through which GBD is expected to operate to produce inequities in health behaviour and ultimately in health outcomes. Such a clear conceptual understanding would also open the way to more operational research, potentially guiding the implementation of interventions targeting GBD with the ultimate intention of reducing health inequities.^275^

Third, the lack of a single tool capturing the different dimensions of experiences and perceptions of GBD is a reflection of how we continue as a research community to lack consensus on how to conceptualise and measure GBD in relation to health outcomes. Given the increased interest in the relationship between GBD and health noted above, the development of a comprehensive tool could prove to be extremely useful and find wide application across settings, once validated. Hence, we encourage future research to invest in the development of such a tool, taking into consideration all relevant dimensions of GBD, including legal and cultural norms, gender roles, decision-making, son preference at birth, healthcare behaviour and health state, violence against women, education and work and money.

### Methodological considerations

Our review has several limitations. First, in line with the scoping review approach, we did not engage in any quality assessment, possibly including studies of lower quality than what would be the case in a systematic review, possibly giving undue importance to some low-quality studies. Second, the analytical strategy, including the definition of the themes, was defined *in itinere* by the authors, as data were extracted, and as such it is subject to arbitrary judgement. Though they have been collectively defined and repeatedly discussed to fit the extracted data with most possible accuracy, our choices might have affected the relative importance of each theme we present. Third, we acknowledge our focus on the methods being used to conceptualise and measure GBD, hence we do not provide any synthesis of the actual effects of GBD on health outcomes, possibly narrowing the scope of our results. Fourth, as mentioned in the introduction, we focused on cisgender women by excluding transgender and non-binary individuals. This choice, made with the perspective of clearly focusing only on GBD, might have limited our capacity to capture the complexity of the forms of GBD. Fifth, our scoping review aims to provide a comprehensive overview of the literature on GBD. However, we recognise that certain themes, such as morbidity and mortality, may be influenced by factors beyond discrimination, such as weak health systems or overburdened healthcare providers. While our review captures what has been reported in the literature, it is important to note that the studies included may not always differentiate between outcomes directly driven by GBD and those resulting from broader structural or systemic issues. Therefore, we caution readers against interpreting all findings as solely indicative of GBD, as some outcomes may reflect complex and intersecting factors.

## Conclusions

This review provides a comprehensive overview of how GBD is measured and conceptualised in the health literature. Our search strategy, which does not prioritise survey measures and is health-related, enables us to link various literature fields, from the psychological constructs of GBD to its consequences on health. The measures include perceptions and behaviours, as well as differential treatment and experiences, and gender-differences in health outcomes (including sex ratio at birth, morbidity and mortality rates) and we synthesised them into 11 themes of GBD, namely healthcare behaviours, health state, son preference at birth, self-declared discrimination, cultural and legal practices, gender roles, access to economic resources, decision-making, education, violence against women, disrespect and abuse during childbirth.

Our results have three important implications: (1) future research on GBD should be explicit about the definition of GBD and the concepts used to measure it in order to deliver clear and actionable messages about how to address it; (2) a consensus should be reached about how GBD is conceptualised and what it encompasses as this could guide future research and policymaking to address GBD and (3) the development of a tool capturing the different dimensions of experiences and perceptions of GBD could be extremely useful and contribute to greater clarity and comparability of research on GBD in the future. Ultimately, improving conceptual clarity and measurement of GBD would help to address GBD and reduce inequities in health outcomes.

## Supplementary material

10.1136/bmjph-2024-002276 online supplemental file 1

10.1136/bmjph-2024-002276 online supplemental file 2

## Data Availability

Data are available upon reasonable request. No data are available.
