# Supplementary material for: Gender-based discrimination towards cisgender women in low- and middle-income countries: a scoping review of definitions and measures used in the health literature
Source: BMJ Public Health. 2026 Jun 30;4(2):e002276. doi: 10.1136/bmjph-2024-002276 (PMC13331126; doi:10.1136/bmjph-2024-002276)
Supplement: online supplemental file 2 [file bmjph-4-2-s002.pdf]

Supplementary Material

Supplementary Table 1 : List of studies per theme and type of study group

see Excel file

Supplementary Table 2: Tools used in composite indices studies

see Excel file

ANNEX 1 DATA SEARCH STRATEGY

PubMed

P

**"Sexism"[Mesh] OR**<br>((Gender*[tiab] OR<br>genital[tiab] OR<br>Sex[tiab] OR<br>Sexism*[tiab] OR<br>Woman*[tiab] OR<br>Women*[tiab] OR<br>Female*[tiab] OR<br>Girl*[tiab])<br>AND<br>(Discrimination*[tiab] OR<br>Bias*[tiab] OR<br>Stigma*[tiab]))	69882

I

**"Health"[Mesh:NoExp] OR**<br>**"Women's Health"[Mesh] OR**<br>**"Disease"[Mesh] OR**<br>health*[tiab] OR<br>medical*[tiab] OR<br>diseases[tiab] OR<br>disorder*[tiab] OR<br>illness*[tiab] OR	7961267
**"Mortality"[Mesh] OR**<br>**"Morbidity"[Mesh] OR**<br>mortalit*[tiab] OR<br>death*[tiab] OR<br>fatalit*[tiab] OR<br>morbidit*[tiab] OR<br>maternal*[tiab] OR<br>maternit*[tiab]	

X

**"Developing Countries"[Mesh] OR**<br>"Developing Country"[tiab] OR<br>"Least Developed Country"[tiab] OR<br>"Less Developed Country"[tiab] OR<br>"Lesser Developed Country"[tiab] OR<br>"Under Developed Country"[tiab] OR<br>"Underdeveloped Country"[tiab] OR<br>"Underserved country"[tiab] OR<br>"deprived country"[tiab] OR<br>"poor country"[tiab] OR<br>"poorer country"[tiab] OR<br>"emerging country"[tiab] OR	2474358

"lmic country"[tiab] OR<br>"lmics country"[tiab] OR<br>"lami country"[tiab] OR<br>"transitional country"[tiab] OR<br>"Developing Countries"[tiab] OR<br>"Least Developed Countries"[tiab] OR<br>"Less Developed Countries"[tiab] OR<br>"Lesser Developed Countries"[tiab] OR<br>"Under Developed Countries"[tiab] OR<br>"Underdeveloped Countries"[tiab] OR<br>"Underserved Countries"[tiab] OR<br>"deprived countries"[tiab] OR<br>"poor countries"[tiab] OR<br>"poorer countries"[tiab] OR<br>"emerging countries"[tiab] OR<br>"lmic countries"[tiab] OR<br>"lmics countries"[tiab] OR<br>"lami countries"[tiab] OR<br>"transitional countries"[tiab] OR<br>"Developing Nation"[tiab] OR<br>"Least Developed nation"[tiab] OR<br>"Less Developed nation"[tiab] OR<br>"Lesser Developed nation"[tiab] OR<br>"Under Developed nation"[tiab] OR<br>"Underdeveloped nation"[tiab] OR<br>"Underserved nation"[tiab] OR<br>"deprived nation"[tiab] OR<br>"poor nation"[tiab] OR<br>"poorer nation"[tiab] OR<br>"emerging nation"[tiab] OR<br>"lmic nation"[tiab] OR<br>"lmics nation"[tiab] OR<br>"lami nation"[tiab] OR<br>"transitional nation"[tiab] OR<br>"Developing Nations"[tiab] OR<br>"Least Developed nations"[tiab] OR<br>"Less Developed nations"[tiab] OR<br>"Lesser Developed nations"[tiab] OR<br>"Under Developed nations"[tiab] OR<br>"Underdeveloped nations"[tiab] OR<br>"Underserved nations"[tiab] OR<br>"deprived nations"[tiab] OR<br>"poor nations"[tiab] OR<br>"poorer nations"[tiab] OR<br>"emerging nations"[tiab] OR<br>"lmic nations"[tiab] OR<br>"lmics nations"[tiab] OR<br>"lami nations"[tiab] OR<br>"transitional nations"[tiab] OR<br>"Developing Population"[tiab] OR<br>"Least Developed population"[tiab] OR<br>"Less Developed population"[tiab] OR<br>"Lesser Developed population"[tiab] OR<br>"Under Developed population"[tiab] OR<br>"Underdeveloped population"[tiab] OR<br>"Underserved population"[tiab] OR<br>"deprived population"[tiab] OR<br>"poor population"[tiab] OR<br>"poorer population"[tiab] OR<br>"emerging population"[tiab] OR	

"lmic population"[tiab] OR<br>"lmics population"[tiab] OR<br>"lami population"[tiab] OR<br>"transitional population"[tiab] OR<br>"Developing Populations"[tiab] OR<br>"Least Developed populations"[tiab] OR<br>"Less Developed populations"[tiab] OR<br>"Lesser Developed populations"[tiab] OR<br>"Under Developed populations"[tiab] OR<br>"Underdeveloped populations"[tiab] OR<br>"Underserved populations"[tiab] OR<br>"deprived populations"[tiab] OR<br>"poor populations"[tiab] OR<br>"poorer populations"[tiab] OR<br>"emerging populations"[tiab] OR<br>"lmic populations"[tiab] OR<br>"lmics populations"[tiab] OR<br>"lami populations"[tiab] OR<br>"transitional populations"[tiab] OR<br>"Developing World"[tiab] OR<br>"Least Developed world"[tiab] OR<br>"Less Developed world"[tiab] OR<br>"Lesser Developed world"[tiab] OR<br>"Under Developed world"[tiab] OR<br>"Underdeveloped world"[tiab] OR<br>"Underserved world"[tiab] OR<br>"deprived world"[tiab] OR<br>"poor world"[tiab] OR<br>"poorer world"[tiab] OR<br>"emerging world"[tiab] OR<br>"lmic world"[tiab] OR<br>"lmics world"[tiab] OR<br>"lami world"[tiab] OR<br>"transitional world"[tiab] OR<br>"Developing economy"[tiab] OR<br>"Least Developed economy"[tiab] OR<br>"Less Developed economy"[tiab] OR<br>"Lesser Developed economy"[tiab] OR<br>"Under Developed economy"[tiab] OR<br>"Underdeveloped economy"[tiab] OR<br>"Underserved economy"[tiab] OR<br>"deprived economy"[tiab] OR<br>"poor economy"[tiab] OR<br>"poorer economy"[tiab] OR<br>"emerging economy"[tiab] OR<br>"lmic economy"[tiab] OR<br>"lmics economy"[tiab] OR<br>"lami economy"[tiab] OR<br>"transitional economy"[tiab] OR<br>"Developing economies"[tiab] OR<br>"Least Developed economies"[tiab] OR<br>"Less Developed economies"[tiab] OR<br>"Lesser Developed economies"[tiab] OR<br>"Under Developed economies"[tiab] OR<br>"Underdeveloped economies"[tiab] OR<br>"Underserved economies"[tiab] OR<br>"deprived economies"[tiab] OR<br>"poor economies"[tiab] OR<br>"poorer economies"[tiab] OR<br>"emerging economies"[tiab] OR	

"lmic economies"[tiab] OR<br>"lmics economies"[tiab] OR<br>"lami economies"[tiab] OR<br>"transitional economies"[tiab] OR<br>"low gross domestic"[tiab] OR<br>"low gross national"[tiab] OR<br>"lower gross domestic"[tiab] OR<br>"lower gross national"[tiab] OR<br>"global south"[tiab] OR<br>"third World"[tiab] OR	
LMIC*[tiab] OR<br>"low and middle income"[tiab] OR<br>"low income"[tiab] OR<br>"middle income"[tiab] OR	
**"Africa South of the Sahara"[Mesh] OR**<br>Subsahara*[tiab] OR	
**Central Africa*[tiab] OR**<br>**Cameroon**[tiab] OR<br>Ubangi Shari*[tiab] OR<br>**Chad**[tiab] OR<br>**Congo**[tiab] OR<br>Zaire[tiab] OR<br>Katanga[tiab] OR<br>Equatorial Guinea*[tiab] OR<br>spanish Guinea*[tiab] OR<br>**Gabon***[tiab] OR<br>**Sao Tome***[tiab] OR	
**East Africa*[tiab] OR**<br>Eastern Africa*[tiab] OR<br>**Burundi**[tiab] OR<br>**Djibouti**[tiab] OR<br>**Eritrea**[tiab] OR<br>**Ethiopia**[tiab] OR<br>**Kenya**[tiab] OR<br>**Rwanda**[tiab] OR<br>**Somalia**[tiab] OR<br>**Sudan**[tiab] OR<br>**Tanzania**[tiab] OR<br>**Uganda**[tiab] OR	
**Southern africa*[tiab] OR**<br>South africa*[tiab] OR<br>**Angola**[tiab] OR<br>**Botswana***[tiab] OR<br>**Eswatini***[tiab] OR<br>**Lesotho***[tiab] OR<br>Basutoland[tiab] OR<br>**Malawi***[tiab] OR<br>**Mozambique***[tiab] OR<br>**Namibia***[tiab] OR<br>**Zambia***[tiab] OR<br>**Zimbabwe***[tiab] OR	
**western africa*[tiab] OR**<br>west africa*[tiab] OR<br>**Benin***[tiab] OR<br>"**Burkina Faso"**[tiab] OR<br>"upper volta"[tiab] OR<br>"burkina fasso"[tiab] OR<br>"**Cabo Verde"**[tiab] OR<br>"cape verde"[tiab] OR<br>"Cote d'Ivoire"[tiab] OR	

"cote d' ivoire"[tiab] OR<br>"cote divoire"[tiab] OR<br>"cote d ivoire"[tiab] OR<br>"ivory coast"[tiab] OR<br>**Gambia***[tiab] OR<br>**Ghana***[tiab] OR<br>**Guinea***[tiab] OR<br>**Liberia***[tiab] OR<br>**Mali***[tiab] OR<br>**Mauritania***[tiab] OR<br>**Niger***[tiab] OR<br>**Senegal***[tiab] OR<br>**Sierra Leone***[tiab] OR<br>**Togo***[tiab] OR	
**"Africa, Northern"[Mesh] OR**<br>north africa*[tiab] OR<br>northern africa*[tiab] OR<br>magreb[tiab] OR<br>maghrib[tiab] OR<br>sahara[tiab] OR<br>Algeria[tiab] OR<br>Egypt*[tiab] OR<br>United Arab Republic*[tiab] OR<br>Libya*[tiab] OR<br>Morocco*[tiab] OR<br>Ifni*[tiab] OR<br>Tunisia[tiab] OR<br>Djibouti[tiab] OR<br>West Bank*[tiab] OR<br>Gaza strip*[tiab] OR	
**Central asia*[tiab] OR**<br>Kazakhstan*[tiab] OR<br>Kyrgyz*[tiab] OR<br>Kirghiz*[tiab] OR<br>Tajikistan[tiab] OR<br>tadjikistan[tiab] OR<br>tadzhik*[tiab] OR<br>Turkmenistan[tiab] OR<br>Uzbekistan[tiab] OR<br>Armenia[tiab] OR<br>Azerbaijan[tiab] OR<br>Georgia[tiab] OR<br>Belarus[tiab] OR<br>Russia[tiab] OR	
**"Asia, Southeastern"[Mesh] OR**<br>South Asia*[tiab] OR<br>Southern Asia*[tiab] OR<br>Borneo*[tiab] OR<br>Brunei*[tiab] OR<br>Cambodia*[tiab] OR<br>Khmer Republic*[tiab] OR<br>Kampuchea*[tiab] OR<br>Indochina*[tiab] OR<br>Indonesia*[tiab] OR<br>Sabah[tiab] OR<br>Sarawak[tiab] OR<br>Laos*[tiab] OR<br>"lao pdr"[tiab] OR<br>"lao people's democratic republic"[tiab] OR	

Malaysia*[tiab] OR<br>Malaya[tiab] OR<br>Mekong*[tiab] OR<br>Myanma*[tiab] OR<br>Philippine*[tiab] OR<br>Philipine*[tiab] OR<br>Singapore*[tiab] OR<br>Thailand*[tiab] OR<br>Siam*[tiab] OR<br>Burma*[tiab] OR<br>Timor Leste*[tiab] OR<br>East Timor*[tiab] OR<br>Vietnam*[tiab] OR<br>Bangladesh*[tiab] OR<br>Bhutan*[tiab] OR<br>Maldives*[tiab] OR	
**west Asia*[tiab] OR**<br>western Asia*[tiab] OR<br>Afghanistan*[tiab] OR<br>Bahrain*[tiab] OR<br>Iran*[tiab] OR<br>Iraq*[tiab] OR<br>Israel*[tiab] OR<br>Jordan*[tiab] OR<br>Kuwait*[tiab] OR<br>Lebanon*[tiab] OR<br>Oman*[tiab] OR<br>Qatar*[tiab] OR<br>Saudi Arabia*[tiab] OR<br>Syria*[tiab] OR<br>Turkey*[tiab] OR<br>Arab Emirate*[tiab] OR<br>Yemen*[tiab] OR<br>Nepal*[tiab] OR<br>Pakistan*[tiab] OR<br>Sri Lanka*[tiab] OR<br>Ceylon*[tiab] OR	
**India*[tiab] OR**<br>Andhra Pradesh*[tiab] OR<br>Arunachal Pradesh*[tiab] OR<br>Assam*[tiab] OR<br>Sikkim*[tiab] OR<br>Bihar*[tiab] OR<br>Chhattisgarh*[tiab] OR<br>Goa[tiab] OR<br>Gujarat*[tiab] OR<br>Haryana*[tiab] OR<br>Himachal Pradesh*[tiab] OR<br>Jammu and Kashmir*[tiab] OR<br>Jharkhand*[tiab] OR<br>Kerala*[tiab] OR<br>Karnataka*[tiab] OR<br>Madhya Pradesh*[tiab] OR<br>Maharashtra*[tiab] OR<br>Manipur*[tiab] OR<br>Meghalaya*[tiab] OR<br>Mizoram*[tiab] OR<br>Nagaland*[tiab] OR<br>Odisha*[tiab] OR<br>Punjab*[tiab] OR	

Rajasthan*[tiab] OR<br>Tamil Nadu*[tiab] OR<br>Telangana*[tiab] OR<br>Tripura*[tiab] OR<br>Uttar Pradesh*[tiab] OR<br>Uttarakhand*[tiab] OR<br>West Bengal*[tw] OR	
**east Asia*[tiab] OR**<br>eastern Asia*[tiab] OR<br>China*[tiab] OR<br>Beijing*[tiab] OR<br>Hong Kong*[tiab] OR<br>Macau*[tiab] OR<br>Tibet*[tiab] OR<br>Korea*[tiab] OR<br>Mongolia*[tiab] OR<br>Fiji*[tiab] OR<br>Micronesia*[tiab] OR<br>Kiribati*[tiab] OR<br>Lao PDR*[tiab] OR<br>Marshall Island*[tiab] OR<br>Papua New Guinea*[tiab] OR<br>"Solomon Island"[tiab] OR<br>"solomon islands"[tiab] OR<br>melanesia[tiab] OR<br>"norfolk island"[tiab] OR<br>Tonga*[tiab] OR<br>Tuvalu*[tiab] OR<br>Vanuatu*[tiab] OR<br>"Samoa"[tiab] OR	
Albania[tiab] OR<br>Bosnia*[tiab] OR<br>Herzegovina*[tiab] OR<br>Moldova*[tiab] OR<br>North Macedonia*[tiab] OR<br>Northern Macedonia*[tiab] OR<br>Montenegro*[tiab] OR<br>Romania[tiab] OR<br>Bulgaria[tiab] OR<br>Serbia[tiab] OR<br>Ukraine[tiab] OR<br>Kosovo*[tiab] OR	Europe & Central Asia
**"Latin America"[Mesh] OR**<br>**"Caribbean Region"[Mesh] OR**<br>Latin America*[tiab] OR<br>Caribbean*[tiab] OR<br>Argentina*[tiab] OR<br>Belize*[tiab] OR<br>Bolivia*[tiab] OR<br>Brazil*[tiab] OR<br>Colombia*[tiab] OR<br>Costa Rica*[tiab] OR<br>Cuba*[tiab] OR<br>Dominica*[tiab] OR<br>Ecuador*[tiab] OR<br>Grenada*[tiab] OR<br>Guatemala*[tiab] OR<br>Guyana*[tiab] OR<br>Honduras*[tiab] OR<br>Haiti*[tiab] OR	

Jamaica*[tiab] OR<br>St. Lucia*[tiab] OR<br>St Lucia*[tiab] OR<br>Saint Lucia*[tiab] OR<br>Mexico*[tiab] OR<br>Nicaragua*[tiab] OR<br>Panama*[tiab] OR<br>Peru*[tiab] OR<br>Paraguay*[tiab] OR<br>El Salvador*[tiab] OR<br>Suriname*[tiab] OR<br>Grenadines[tiab] OR<br>"saint vincent"[tiab] OR<br>"st vincent"[tiab] OR<br>Venezuela[tiab] OR<br>Antigua[tiab] OR<br>Barbuda[tiab] OR<br>Bahama*[tiab] OR<br>Barbados[tiab] OR<br>Guadeloupe[tiab] OR<br>Martinique[tiab] OR<br>"Puerto Rico"[tiab] OR<br>Saint Kitts*[tiab] OR<br>St Kitts*[tiab] OR<br>Trinidad[tiab] OR<br>Tobago[tiab] OR<br>Virgin Island*[tiab] OR<br>Aruba[tiab] OR<br>Curacao[tiab] OR<br>"Sint Maarten"[tiab] OR<br>"West Indies"[tiab]	

P AND I AND X: 9654

Filter from 1985: 9586

Web of Science Core Collection

Records number	Date
7242	08.10.2021

P

("Gender*" OR<br>"genital" OR<br>"Sex" OR<br>"Sexism*" OR<br>"Woman*" OR<br>"Women*" OR<br>"Female*" OR<br>"Girl*")<br>AND<br>("Discrimination*" OR<br>"Bias*" OR<br>"Stigma*")	

I

"health*" OR<br>"medical*" OR<br>"diseases" OR<br>"disorder*" OR<br>"illness*" OR<br>"mortalit*" OR<br>"death*" OR<br>"fatalit*" OR<br>"morbidit*" OR<br>"maternal*" OR<br>"maternit*"	

X

"Developing Country" OR<br>"Least Developed Country" OR<br>"Less Developed Country" OR<br>"Lesser Developed Country" OR<br>"Under Developed Country" OR<br>"Underdeveloped Country" OR<br>"Underserved country" OR<br>"deprived country" OR<br>"poor country" OR<br>"poorer country" OR<br>"emerging country" OR<br>"lmic country" OR<br>"lmics country" OR<br>"lami country" OR<br>"transitional country" OR<br>"Developing Countries" OR<br>"Least Developed Countries" OR<br>"Less Developed Countries" OR<br>"Lesser Developed Countries" OR<br>"Under Developed Countries" OR<br>"Underdeveloped Countries" OR<br>"Underserved Countries" OR<br>"deprived countries" OR<br>"poor countries" OR<br>"poorer countries" OR<br>"emerging countries" OR<br>"lmic countries" OR<br>"lmics countries" OR<br>"lami countries" OR	

"transitional countries" OR<br>"Developing Nation" OR<br>"Least Developed nation" OR<br>"Less Developed nation" OR<br>"Lesser Developed nation" OR<br>"Under Developed nation" OR<br>"Underdeveloped nation" OR<br>"Underserved nation" OR<br>"deprived nation" OR<br>"poor nation" OR<br>"poorer nation" OR<br>"emerging nation" OR<br>"lmic nation" OR<br>"lmics nation" OR<br>"lami nation" OR<br>"transitional nation" OR<br>"Developing Nations" OR<br>"Least Developed nations" OR<br>"Less Developed nations" OR<br>"Lesser Developed nations" OR<br>"Under Developed nations" OR<br>"Underdeveloped nations" OR<br>"Underserved nations" OR<br>"deprived nations" OR<br>"poor nations" OR<br>"poorer nations" OR<br>"emerging nations" OR<br>"lmic nations" OR<br>"lmics nations" OR<br>"lami nations" OR<br>"transitional nations" OR<br>"Developing Population" OR<br>"Least Developed population" OR<br>"Less Developed population" OR<br>"Lesser Developed population" OR<br>"Under Developed population" OR<br>"Underdeveloped population" OR<br>"Underserved population" OR<br>"deprived population" OR<br>"poor population" OR<br>"poorer population" OR<br>"emerging population" OR<br>"lmic population" OR<br>"lmics population" OR<br>"lami population" OR<br>"transitional population" OR<br>"Developing Populations" OR<br>"Least Developed populations" OR<br>"Less Developed populations" OR<br>"Lesser Developed populations" OR<br>"Under Developed populations" OR<br>"Underdeveloped populations" OR<br>"Underserved populations" OR<br>"deprived populations" OR<br>"poor populations" OR<br>"poorer populations" OR<br>"emerging populations" OR<br>"lmic populations" OR<br>"lmics populations" OR<br>"lami populations" OR	

"transitional populations" OR<br>"Developing World" OR<br>"Least Developed world" OR<br>"Less Developed world" OR<br>"Lesser Developed world" OR<br>"Under Developed world" OR<br>"Underdeveloped world" OR<br>"Underserved world" OR<br>"deprived world" OR<br>"poor world" OR<br>"poorer world" OR<br>"emerging world" OR<br>"lmic world" OR<br>"lmics world" OR<br>"lami world" OR<br>"transitional world" OR<br>"Developing economy" OR<br>"Least Developed economy" OR<br>"Less Developed economy" OR<br>"Lesser Developed economy" OR<br>"Under Developed economy" OR<br>"Underdeveloped economy" OR<br>"Underserved economy" OR<br>"deprived economy" OR<br>"poor economy" OR<br>"poorer economy" OR<br>"emerging economy" OR<br>"lmic economy" OR<br>"lmics economy" OR<br>"lami economy" OR<br>"transitional economy" OR<br>"Developing economies" OR<br>"Least Developed economies" OR<br>"Less Developed economies" OR<br>"Lesser Developed economies" OR<br>"Under Developed economies" OR<br>"Underdeveloped economies" OR<br>"Underserved economies" OR<br>"deprived economies" OR<br>"poor economies" OR<br>"poorer economies" OR<br>"emerging economies" OR<br>"lmic economies" OR<br>"lmics economies" OR<br>"lami economies" OR<br>"transitional economies" OR<br>"low gross domestic" OR<br>"low gross national" OR<br>"lower gross domestic" OR<br>"lower gross national" OR<br>"global south" OR<br>"third World" OR<br>"LMIC*" OR<br>"low and middle income" OR<br>"low income" OR<br>"middle income" OR<br>"Subsahara*" OR<br>"Central Africa*" OR<br>"Cameroon" OR<br>"Ubangi Shari*" OR	

"Chad" OR<br>"Congo" OR<br>"Zaire" OR<br>"Katanga" OR<br>"Equatorial Guinea*" OR<br>"spanish Guinea*" OR<br>"Gabon*" OR<br>"Sao Tome*" OR<br>"East Africa*" OR<br>"Eastern Africa*" OR<br>"Burundi" OR<br>"Djibouti" OR<br>"Eritrea" OR<br>"Ethiopia" OR<br>"Kenya" OR<br>"Rwanda" OR<br>"Somalia" OR<br>"Sudan" OR<br>"Tanzania" OR<br>"Uganda" OR<br>"Southern africa*" OR<br>"South africa*" OR<br>"Angola" OR<br>"Botswana*" OR<br>"Eswatini*" OR<br>"Lesotho*" OR<br>"Basutoland" OR<br>"Malawi*" OR<br>"Mozambique*" OR<br>"Namibia*" OR<br>"Zambia*" OR<br>"Zimbabwe*" OR<br>"western africa*" OR<br>"west africa*" OR<br>"Benin*" OR<br>"Burkina Faso" OR<br>"upper volta" OR<br>"burkina fasso" OR<br>"Cabo Verde" OR<br>"cape verde" OR<br>"Cote dIvoire" OR<br>"cote d' ivoire" OR<br>"cote divoire" OR<br>"cote d ivoire" OR<br>"ivory coast" OR<br>"Gambia*" OR<br>"Ghana*" OR<br>"Guinea*" OR<br>"Liberia*" OR<br>"Mali*" OR<br>"Mauritania*" OR<br>"Niger*" OR<br>"Senegal*" OR<br>"Sierra Leone*" OR<br>"Togo*" OR<br>"north africa*" OR<br>"northern africa*" OR<br>"magreb" OR<br>"maghrib" OR<br>"sahara" OR	

"Algeria" OR<br>"Egypt*" OR<br>"United Arab Republic*" OR<br>"Libya*" OR<br>"Morocco*" OR<br>"Ifni*" OR<br>"Tunisia" OR<br>"Djibouti" OR<br>"West Bank*" OR<br>"Gaza strip*" OR<br>"Central asia*" OR<br>"Kazakhstan*" OR<br>"Kyrgyz*" OR<br>"Kirghiz*" OR<br>"Tajikistan" OR<br>"tadjikistan" OR<br>"tadzhik*" OR<br>"Turkmenistan" OR<br>"Uzbekistan" OR<br>"Armenia" OR<br>"Azerbaijan" OR<br>"Georgia" OR<br>"Belarus" OR<br>"Russia" OR<br>"South Asia*" OR<br>"Southern Asia*" OR<br>"Borneo*" OR<br>"Brunei*" OR<br>"Cambodia*" OR<br>"Khmer Republic*" OR<br>"Kampuchea*" OR<br>"Indochina*" OR<br>"Indonesia*" OR<br>"Sabah" OR<br>"Sarawak" OR<br>"Laos*" OR<br>"lao pdr" OR<br>"lao peoples democratic republic" OR<br>"Malaysia*" OR<br>"Malaya" OR<br>"Mekong*" OR<br>"Myanma*" OR<br>"Philippine*" OR<br>"Philipine*" OR<br>"Singapore*" OR<br>"Thailand*" OR<br>"Siam*" OR<br>"Burma*" OR<br>"Timor Leste*" OR<br>"East Timor*" OR<br>"Vietnam*" OR<br>"Bangladesh*" OR<br>"Bhutan*" OR<br>"Maldives*" OR<br>"west Asia*" OR<br>"western Asia*" OR<br>"Afghanistan*" OR<br>"Bahrain*" OR<br>"Iran*" OR<br>"Iraq*" OR	

"Israel*" OR<br>"Jordan*" OR<br>"Kuwait*" OR<br>"Lebanon*" OR<br>"Oman*" OR<br>"Qatar*" OR<br>"Saudi Arabia*" OR<br>"Syria*" OR<br>"Turkey*" OR<br>"Arab Emirate*" OR<br>"Yemen*" OR<br>"Nepal*" OR<br>"Pakistan*" OR<br>"Sri Lanka*" OR<br>"Ceylon*" OR<br>"India*" OR<br>"Andhra Pradesh*" OR<br>"Arunachal Pradesh*" OR<br>"Assam*" OR<br>"Sikkim*" OR<br>"Bihar*" OR<br>"Chhattisgarh*" OR<br>"Goa" OR<br>"Gujarat*" OR<br>"Haryana*" OR<br>"Himachal Pradesh*" OR<br>"Jammu and Kashmir*" OR<br>"Jharkhand*" OR<br>"Kerala*" OR<br>"Karnataka*" OR<br>"Madhya Pradesh*" OR<br>"Maharashtra*" OR<br>"Manipur*" OR<br>"Meghalaya*" OR<br>"Mizoram*" OR<br>"Nagaland*" OR<br>"Odisha*" OR<br>"Punjab*" OR<br>"Rajasthan*" OR<br>"Tamil Nadu*" OR<br>"Telangana*" OR<br>"Tripura*" OR<br>"Uttar Pradesh*" OR<br>"Uttarakhand*" OR<br>"West Bengal*" OR<br>"east Asia*" OR<br>"eastern Asia*" OR<br>"China*" OR<br>"Beijing*" OR<br>"Hong Kong*" OR<br>"Macau*" OR<br>"Tibet*" OR<br>"Korea*" OR<br>"Mongolia*" OR<br>"Fiji*" OR<br>"Micronesia*" OR<br>"Kiribati*" OR<br>"Lao PDR*" OR<br>"Marshall Island*" OR<br>"Papua New Guinea*" OR	

"Solomon Island" OR<br>"solomon islands" OR<br>"melanesia" OR<br>"norfolk island" OR<br>"Tonga*" OR<br>"Tuvalu*" OR<br>"Vanuatu*" OR<br>"Samoa" OR<br>"Albania" OR<br>"Bosnia*" OR<br>"Herzegovina*" OR<br>"Moldova*" OR<br>"North Macedonia*" OR<br>"Northern Macedonia*" OR<br>"Montenegro*" OR<br>"Romania" OR<br>"Bulgaria" OR<br>"Serbia" OR<br>"Ukraine" OR<br>"Kosovo*" OR<br>"Latin America*" OR<br>"Caribbean*" OR<br>"Argentina*" OR<br>"Belize*" OR<br>"Bolivia*" OR<br>"Brazil*" OR<br>"Colombia*" OR<br>"Costa Rica*" OR<br>"Cuba*" OR<br>"Dominica*" OR<br>"Ecuador*" OR<br>"Grenada*" OR<br>"Guatemala*" OR<br>"Guyana*" OR<br>"Honduras*" OR<br>"Haiti*" OR<br>"Jamaica*" OR<br>"St. Lucia*" OR<br>"St Lucia*" OR<br>"Saint Lucia*" OR<br>"Mexico*" OR<br>"Nicaragua*" OR<br>"Panama*" OR<br>"Peru*" OR<br>"Paraguay*" OR<br>"El Salvador*" OR<br>"Suriname*" OR<br>"Grenadines" OR<br>"saint vincent" OR<br>"st vincent" OR<br>"Venezuela" OR<br>"Antigua" OR<br>"Barbuda" OR<br>"Bahama*" OR<br>"Barbados" OR<br>"Guadeloupe" OR<br>"Martinique" OR<br>"Puerto Rico" OR<br>"Saint Kitts*" OR<br>"St Kitts*" OR	

"Trinidad" OR<br>"Tobago" OR<br>"Virgin Island*" OR<br>"Aruba" OR<br>"Curacao" OR<br>"Sint Maarten" OR<br>"West Indies"	

P (78783)

TI=(("Gender*" OR "genital" OR "Sex" OR "Sexism*" OR "Woman*" OR "Women*" OR "Female*" OR "Girl*") AND ("Discrimination*" OR "Bias*" OR "Stigma*"))

OR

AB=(("Gender*" OR "genital" OR "Sex" OR "Sexism*" OR "Woman*" OR "Women*" OR "Female*" OR "Girl*") AND ("Discrimination*" OR "Bias*" OR "Stigma*"))

I (6647113)

TI=("health*" OR "medical*" OR "diseases" OR "disorder*" OR "illness*" OR "mortalit*" OR "death*" OR "fatalit*" OR "morbidit*" OR "maternal*" OR "maternit*")

OR

AB=("health*" OR "medical*" OR "diseases" OR "disorder*" OR "illness*" OR "mortalit*" OR "death*" OR "fatalit*" OR "morbidit*" OR "maternal*" OR "maternit*")

X (3946873)

TI=("Developing Country" OR "Least Developed Country" OR "Less Developed Country" OR "Lesser Developed Country" OR "Under Developed Country" OR "Underdeveloped Country" OR "Underserved country" OR "deprived country" OR "poor country" OR "poorer country" OR "emerging country" OR "lmic country" OR "lmics country" OR "lami country" OR "transitional country" OR "Developing Countries" OR "Least Developed Countries" OR "Less Developed Countries" OR "Lesser Developed Countries" OR "Under Developed Countries" OR "Underdeveloped Countries" OR "Underserved Countries" OR "deprived countries" OR "poor countries" OR "poorer countries" OR "emerging countries" OR "lmic countries" OR "lmics countries" OR "lami countries" OR "transitional countries" OR "Developing Nation" OR "Least Developed nation" OR "Less Developed nation" OR "Lesser Developed nation" OR "Under Developed nation" OR "Underdeveloped nation" OR "Underserved nation" OR "deprived nation" OR "poor nation" OR "poorer nation" OR "emerging nation" OR "lmic nation" OR "lmics nation" OR "lami nation" OR "transitional nation" OR "Developing Nations" OR "Least Developed nations" OR "Less Developed nations" OR "Lesser Developed nations" OR "Under Developed nations" OR "Underdeveloped nations" OR "Underserved nations" OR "deprived nations" OR "poor nations" OR "poorer nations" OR "emerging nations" OR "lmic nations" OR "lmics nations" OR "lami nations" OR "transitional nations" OR "Developing Population" OR "Least Developed population" OR "Less Developed population" OR "Lesser Developed population" OR "Under Developed population" OR "Underdeveloped population" OR "Underserved population" OR "deprived population" OR "poor population" OR "poorer population" OR "emerging population" OR "lmic population" OR "lmics population" OR "lami population" OR "transitional population" OR "Developing Populations" OR "Least Developed populations" OR "Less Developed populations" OR "Lesser Developed populations" OR "Under Developed populations" OR "Underdeveloped populations" OR "Underserved populations" OR "deprived populations" OR "poor populations" OR "poorer populations" OR "emerging populations" OR "lmic populations" OR "lmics populations" OR "lami populations" OR "transitional populations" OR "Developing World" OR "Least Developed world" OR "Less Developed world" OR "Lesser Developed world" OR "Under Developed world" OR "Underdeveloped world" OR "Underserved world" OR "deprived world" OR "poor world" OR "poorer world" OR "emerging world" OR "lmic world" OR "lmics world"

OR "lami world" OR "transitional world" OR "Developing economy" OR "Least Developed economy" OR "Less Developed economy" OR "Lesser Developed economy" OR "Under Developed economy" OR "Underdeveloped economy" OR "Underserved economy" OR "deprived economy" OR "poor economy" OR "poorer economy" OR "emerging economy" OR "lmic economy" OR "lmics economy" OR "lami economy" OR "transitional economy" OR "Developing economies" OR "Least Developed economies" OR "Less Developed economies" OR "Lesser Developed economies" OR "Under Developed economies" OR "Underdeveloped economies" OR "Underserved economies" OR "deprived economies" OR "poor economies" OR "poorer economies" OR "emerging economies" OR "lmic economies" OR "lmics economies" OR "lami economies" OR "transitional economies" OR "low gross domestic" OR "low gross national" OR "lower gross domestic" OR "lower gross national" OR "global south" OR "third World" OR "LMIC*" OR "low and middle income" OR "low income" OR "middle income" OR "Subsahara*" OR "Central Africa*" OR "Cameroon" OR "Ubangi Shari*" OR "Chad" OR "Congo" OR "Zaire" OR "Katanga" OR "Equatorial Guinea*" OR "spanish Guinea*" OR "Gabon*" OR "Sao Tome*" OR "East Africa*" OR "Eastern Africa*" OR "Burundi" OR "Djibouti" OR "Eritrea" OR "Ethiopia" OR "Kenya" OR "Rwanda" OR "Somalia" OR "Sudan" OR "Tanzania" OR "Uganda" OR "Southern africa*" OR "South africa*" OR "Angola" OR "Botswana*" OR "Eswatini*" OR "Lesotho*" OR "Basutoland" OR "Malawi*" OR "Mozambique*" OR "Namibia*" OR "Zambia*" OR "Zimbabwe*" OR "western africa*" OR "west africa*" OR "Benin*" OR "Burkina Faso" OR "upper volta" OR "burkina fasso" OR "Cabo Verde" OR "cape verde" OR "Cote dIvoire" OR "cote d' ivoire" OR "cote divoire" OR "cote d ivoire" OR "ivory coast" OR "Gambia*" OR "Ghana*" OR "Guinea*" OR "Liberia*" OR "Mali*" OR "Mauritania*" OR "Niger*" OR "Senegal*" OR "Sierra Leone*" OR "Togo*" OR "north africa*" OR "northern africa*" OR "magreb" OR "maghrib" OR "sahara" OR "Algeria" OR "Egypt*" OR "United Arab Republic*" OR "Libya*" OR "Morocco*" OR "Ifni*" OR "Tunisia" OR "Djibouti" OR "West Bank*" OR "Gaza strip*" OR "Central asia*" OR "Kazakhstan*" OR "Kyrgyz*" OR "Kirghiz*" OR "Tajikistan" OR "tadjikistan" OR "tadzhik*" OR "Turkmenistan" OR "Uzbekistan" OR "Armenia" OR "Azerbaijan" OR "Georgia" OR "Belarus" OR "Russia" OR "South Asia*" OR "Southern Asia*" OR "Borneo*" OR "Brunei*" OR "Cambodia*" OR "Khmer Republic*" OR "Kampuchea*" OR "Indochina*" OR "Indonesia*" OR "Sabah" OR "Sarawak" OR "Laos*" OR "lao pdr" OR "lao peoples democratic republic" OR "Malaysia*" OR "Malaya" OR "Mekong*" OR "Myanma*" OR "Philippine*" OR "Philipine*" OR "Singapore*" OR "Thailand*" OR "Siam*" OR "Burma*" OR "Timor Leste*" OR "East Timor*" OR "Vietnam*" OR "Bangladesh*" OR "Bhutan*" OR "Maldives*" OR "west Asia*" OR "western Asia*" OR "Afghanistan*" OR "Bahrain*" OR "Iran*" OR "Iraq*" OR "Israel*" OR "Jordan*" OR "Kuwait*" OR "Lebanon*" OR "Oman*" OR "Qatar*" OR "Saudi Arabia*" OR "Syria*" OR "Turkey*" OR "Arab Emirate*" OR "Yemen*" OR "Nepal*" OR "Pakistan*" OR "Sri Lanka*" OR "Ceylon*" OR "India*" OR "Andhra Pradesh*" OR "Arunachal Pradesh*" OR "Assam*" OR "Sikkim*" OR "Bihar*" OR "Chhattisgarh*" OR "Goa" OR "Gujarat*" OR "Haryana*" OR "Himachal Pradesh*" OR "Jammu and Kashmir*" OR "Jharkhand*" OR "Kerala*" OR "Karnataka*" OR "Madhya Pradesh*" OR "Maharashtra*" OR "Manipur*" OR "Meghalaya*" OR "Mizoram*" OR "Nagaland*" OR "Odisha*" OR "Punjab*" OR "Rajasthan*" OR "Tamil Nadu*" OR "Telangana*" OR "Tripura*" OR "Uttar Pradesh*" OR "Uttarakhand*" OR "West Bengal*" OR "east Asia*" OR "eastern Asia*" OR "China*" OR "Beijing*" OR "Hong Kong*" OR "Macau*" OR "Tibet*" OR "Korea*" OR "Mongolia*" OR "Fiji*" OR "Micronesia*" OR "Kiribati*" OR "Lao PDR*" OR "Marshall Island*" OR "Papua New Guinea*" OR "Solomon Island" OR "solomon islands" OR "melanesia" OR "norfolk island" OR "Tonga*" OR "Tuvalu*" OR "Vanuatu*" OR "Samoa" OR "Albania" OR "Bosnia*" OR "Herzegovina*" OR "Moldova*" OR "North Macedonia*" OR "Northern Macedonia*" OR "Montenegro*" OR "Romania" OR "Bulgaria" OR "Serbia" OR "Ukraine" OR "Kosovo*" OR "Latin America*" OR "Caribbean*" OR "Argentina*" OR "Belize*" OR "Bolivia*" OR "Brazil*" OR "Colombia*" OR "Costa Rica*" OR "Cuba*" OR "Dominica*" OR "Ecuador*" OR "Grenada*" OR "Guatemala*" OR "Guyana*" OR "Honduras*" OR "Haiti*" OR "Jamaica*" OR "St. Lucia*" OR "St Lucia*" OR "Saint Lucia*" OR "Mexico*" OR "Nicaragua*" OR "Panama*" OR "Peru*" OR "Paraguay*" OR "El Salvador*" OR "Suriname*" OR "Grenadines" OR "saint vincent" OR "st vincent" OR "Venezuela" OR "Antigua" OR "Barbuda" OR "Bahama*" OR "Barbados" OR "Guadeloupe" OR "Martinique" OR "Puerto Rico" OR "Saint Kitts*" OR "St Kitts*" OR "Trinidad" OR "Tobago" OR "Virgin Island*" OR "Aruba" OR "Curacao" OR "Sint Maarten" OR "West Indies")

OR

AB=("Developing Country" OR "Least Developed Country" OR "Less Developed Country" OR "Lesser Developed Country" OR "Under Developed Country" OR "Underdeveloped Country" OR "Underserved country" OR

"deprived country" OR "poor country" OR "poorer country" OR "emerging country" OR "lmic country" OR "lmics country" OR "lami country" OR "transitional country" OR "Developing Countries" OR "Least Developed Countries" OR "Less Developed Countries" OR "Lesser Developed Countries" OR "Under Developed Countries" OR "Underdeveloped Countries" OR "Underserved Countries" OR "deprived countries" OR "poor countries" OR "poorer countries" OR "emerging countries" OR "lmic countries" OR "lmics countries" OR "lami countries" OR "transitional countries" OR "Developing Nation" OR "Least Developed nation" OR "Less Developed nation" OR "Lesser Developed nation" OR "Under Developed nation" OR "Underdeveloped nation" OR "Underserved nation" OR "deprived nation" OR "poor nation" OR "poorer nation" OR "emerging nation" OR "lmic nation" OR "lmics nation" OR "lami nation" OR "transitional nation" OR "Developing Nations" OR "Least Developed nations" OR "Less Developed nations" OR "Lesser Developed nations" OR "Under Developed nations" OR "Underdeveloped nations" OR "Underserved nations" OR "deprived nations" OR "poor nations" OR "poorer nations" OR "emerging nations" OR "lmic nations" OR "lmics nations" OR "lami nations" OR "transitional nations" OR "Developing Population" OR "Least Developed population" OR "Less Developed population" OR "Lesser Developed population" OR "Under Developed population" OR "Underdeveloped population" OR "Underserved population" OR "deprived population" OR "poor population" OR "poorer population" OR "emerging population" OR "lmic population" OR "lmics population" OR "lami population" OR "transitional population" OR "Developing Populations" OR "Least Developed populations" OR "Less Developed populations" OR "Lesser Developed populations" OR "Under Developed populations" OR "Underdeveloped populations" OR "Underserved populations" OR "deprived populations" OR "poor populations" OR "poorer populations" OR "emerging populations" OR "lmic populations" OR "lmics populations" OR "lami populations" OR "transitional populations" OR "Developing World" OR "Least Developed world" OR "Less Developed world" OR "Lesser Developed world" OR "Under Developed world" OR "Underdeveloped world" OR "Underserved world" OR "deprived world" OR "poor world" OR "poorer world" OR "emerging world" OR "lmic world" OR "lmics world" OR "lami world" OR "transitional world" OR "Developing economy" OR "Least Developed economy" OR "Less Developed economy" OR "Lesser Developed economy" OR "Under Developed economy" OR "Underdeveloped economy" OR "Underserved economy" OR "deprived economy" OR "poor economy" OR "poorer economy" OR "emerging economy" OR "lmic economy" OR "lmics economy" OR "lami economy" OR "transitional economy" OR "Developing economies" OR "Least Developed economies" OR "Less Developed economies" OR "Lesser Developed economies" OR "Under Developed economies" OR "Underdeveloped economies" OR "Underserved economies" OR "deprived economies" OR "poor economies" OR "poorer economies" OR "emerging economies" OR "lmic economies" OR "lmics economies" OR "lami economies" OR "transitional economies" OR "low gross domestic" OR "low gross national" OR "lower gross domestic" OR "lower gross national" OR "global south" OR "third World" OR "LMIC*" OR "low and middle income" OR "low income" OR "middle income" OR "Subsahara*" OR "Central Africa*" OR "Cameroon" OR "Ubangi Shari*" OR "Chad" OR "Congo" OR "Zaire" OR "Katanga" OR "Equatorial Guinea*" OR "spanish Guinea*" OR "Gabon*" OR "Sao Tome*" OR "East Africa*" OR "Eastern Africa*" OR "Burundi" OR "Djibouti" OR "Eritrea" OR "Ethiopia" OR "Kenya" OR "Rwanda" OR "Somalia" OR "Sudan" OR "Tanzania" OR "Uganda" OR "Southern africa*" OR "South africa*" OR "Angola" OR "Botswana*" OR "Eswatini*" OR "Lesotho*" OR "Basutoland" OR "Malawi*" OR "Mozambique*" OR "Namibia*" OR "Zambia*" OR "Zimbabwe*" OR "western africa*" OR "west africa*" OR "Benin*" OR "Burkina Faso" OR "upper volta" OR "burkina fasso" OR "Cabo Verde" OR "cape verde" OR "Cote dIvoire" OR "cote d' ivoire" OR "cote divoire" OR "cote d ivoire" OR "ivory coast" OR "Gambia*" OR "Ghana*" OR "Guinea*" OR "Liberia*" OR "Mali*" OR "Mauritania*" OR "Niger*" OR "Senegal*" OR "Sierra Leone*" OR "Togo*" OR "north africa*" OR "northern africa*" OR "magreb" OR "maghrib" OR "sahara" OR "Algeria" OR "Egypt*" OR "United Arab Republic*" OR "Libya*" OR "Morocco*" OR "Ifni*" OR "Tunisia" OR "Djibouti" OR "West Bank*" OR "Gaza strip*" OR "Central asia*" OR "Kazakhstan*" OR "Kyrgyz*" OR "Kirghiz*" OR "Tajikistan" OR "tadjikistan" OR "tadzhik*" OR "Turkmenistan" OR "Uzbekistan" OR "Armenia" OR "Azerbaijan" OR "Georgia" OR "Belarus" OR "Russia" OR "South Asia*" OR "Southern Asia*" OR "Borneo*" OR "Brunei*" OR "Cambodia*" OR "Khmer Republic*" OR "Kampuchea*" OR "Indochina*" OR "Indonesia*" OR "Sabah" OR "Sarawak" OR "Laos*" OR "lao pdr" OR "lao peoples democratic republic" OR "Malaysia*" OR "Malaya" OR "Mekong*" OR "Myanma*" OR "Philippine*" OR "Philipine*" OR "Singapore*" OR "Thailand*" OR "Siam*" OR "Burma*" OR "Timor Leste*" OR "East Timor*" OR "Vietnam*" OR "Bangladesh*" OR "Bhutan*" OR "Maldives*" OR "west Asia*" OR "western Asia*" OR "Afghanistan*" OR "Bahrain*" OR "Iran*" OR "Iraq*" OR "Israel*" OR "Jordan*" OR "Kuwait*" OR "Lebanon*" OR "Oman*" OR "Qatar*" OR "Saudi Arabia*" OR "Syria*" OR "Turkey*" OR "Arab Emirate*" OR

"Yemen*" OR "Nepal*" OR "Pakistan*" OR "Sri Lanka*" OR "Ceylon*" OR "India*" OR "Andhra Pradesh*" OR "Arunachal Pradesh*" OR "Assam*" OR "Sikkim*" OR "Bihar*" OR "Chhattisgarh*" OR "Goa" OR "Gujarat*" OR "Haryana*" OR "Himachal Pradesh*" OR "Jammu and Kashmir*" OR "Jharkhand*" OR "Kerala*" OR "Karnataka*" OR "Madhya Pradesh*" OR "Maharashtra*" OR "Manipur*" OR "Meghalaya*" OR "Mizoram*" OR "Nagaland*" OR "Odisha*" OR "Punjab*" OR "Rajasthan*" OR "Tamil Nadu*" OR "Telangana*" OR "Tripura*" OR "Uttar Pradesh*" OR "Uttarakhand*" OR "West Bengal*" OR "east Asia*" OR "eastern Asia*" OR "China*" OR "Beijing*" OR "Hong Kong*" OR "Macau*" OR "Tibet*" OR "Korea*" OR "Mongolia*" OR "Fiji*" OR "Micronesia*" OR "Kiribati*" OR "Lao PDR*" OR "Marshall Island*" OR "Papua New Guinea*" OR "Solomon Island" OR "solomon islands" OR "melanesia" OR "norfolk island" OR "Tonga*" OR "Tuvalu*" OR "Vanuatu*" OR "Samoa" OR "Albania" OR "Bosnia*" OR "Herzegovina*" OR "Moldova*" OR "North Macedonia*" OR "Northern Macedonia*" OR "Montenegro*" OR "Romania" OR "Bulgaria" OR "Serbia" OR "Ukraine" OR "Kosovo*" OR "Latin America*" OR "Caribbean*" OR "Argentina*" OR "Belize*" OR "Bolivia*" OR "Brazil*" OR "Colombia*" OR "Costa Rica*" OR "Cuba*" OR "Dominica*" OR "Ecuador*" OR "Grenada*" OR "Guatemala*" OR "Guyana*" OR "Honduras*" OR "Haiti*" OR "Jamaica*" OR "St. Lucia*" OR "St Lucia*" OR "Saint Lucia*" OR "Mexico*" OR "Nicaragua*" OR "Panama*" OR "Peru*" OR "Paraguay*" OR "El Salvador*" OR "Suriname*" OR "Grenadines" OR "saint vincent" OR "st vincent" OR "Venezuela" OR "Antigua" OR "Barbuda" OR "Bahama*" OR "Barbados" OR "Guadeloupe" OR "Martinique" OR "Puerto Rico" OR "Saint Kitts*" OR "St Kitts*" OR "Trinidad" OR "Tobago" OR "Virgin Island*" OR "Aruba" OR "Curacao" OR "Sint Maarten" OR "West Indies")

P AND I AND X: 7243

Filter from 1985: 7242

CINAHL

P

("Gender*" OR<br>"genital" OR<br>"Sex" OR<br>"Sexism*" OR<br>"Woman*" OR<br>"Women*" OR<br>"Female*" OR<br>"Girl*")<br>AND<br>("Discrimination*" OR<br>"Bias*" OR<br>"Stigma*")	24255

I

"health*" OR<br>"medical*" OR<br>"diseases" OR<br>"disorder*" OR<br>"illness*" OR<br>"mortalit*" OR<br>"death*" OR<br>"fatalit*" OR<br>"morbidit*" OR<br>"maternal*" OR<br>"maternit*"	2215195

X

"Developing Country" OR<br>"Least Developed Country" OR<br>"Less Developed Country" OR<br>"Lesser Developed Country" OR<br>"Under Developed Country" OR<br>"Underdeveloped Country" OR<br>"Underserved country" OR<br>"deprived country" OR<br>"poor country" OR<br>"poorer country" OR<br>"emerging country" OR<br>"lmic country" OR<br>"lmics country" OR<br>"lami country" OR<br>"transitional country" OR<br>"Developing Countries" OR<br>"Least Developed Countries" OR<br>"Less Developed Countries" OR<br>"Lesser Developed Countries" OR<br>"Under Developed Countries" OR<br>"Underdeveloped Countries" OR<br>"Underserved Countries" OR<br>"deprived countries" OR<br>"poor countries" OR<br>"poorer countries" OR<br>"emerging countries" OR<br>"lmic countries" OR<br>"lmics countries" OR<br>"lami countries" OR<br>"transitional countries" OR<br>"Developing Nation" OR	505184

"Least Developed nation" OR<br>"Less Developed nation" OR<br>"Lesser Developed nation" OR<br>"Under Developed nation" OR<br>"Underdeveloped nation" OR<br>"Underserved nation" OR<br>"deprived nation" OR<br>"poor nation" OR<br>"poorer nation" OR<br>"emerging nation" OR<br>"lmic nation" OR<br>"lmics nation" OR<br>"lami nation" OR<br>"transitional nation" OR<br>"Developing Nations" OR<br>"Least Developed nations" OR<br>"Less Developed nations" OR<br>"Lesser Developed nations" OR<br>"Under Developed nations" OR<br>"Underdeveloped nations" OR<br>"Underserved nations" OR<br>"deprived nations" OR<br>"poor nations" OR<br>"poorer nations" OR<br>"emerging nations" OR<br>"lmic nations" OR<br>"lmics nations" OR<br>"lami nations" OR<br>"transitional nations" OR<br>"Developing Population" OR<br>"Least Developed population" OR<br>"Less Developed population" OR<br>"Lesser Developed population" OR<br>"Under Developed population" OR<br>"Underdeveloped population" OR<br>"Underserved population" OR<br>"deprived population" OR<br>"poor population" OR<br>"poorer population" OR<br>"emerging population" OR<br>"lmic population" OR<br>"lmics population" OR<br>"lami population" OR<br>"transitional population" OR<br>"Developing Populations" OR<br>"Least Developed populations" OR<br>"Less Developed populations" OR<br>"Lesser Developed populations" OR<br>"Under Developed populations" OR<br>"Underdeveloped populations" OR<br>"Underserved populations" OR<br>"deprived populations" OR<br>"poor populations" OR<br>"poorer populations" OR<br>"emerging populations" OR<br>"lmic populations" OR<br>"lmics populations" OR<br>"lami populations" OR<br>"transitional populations" OR<br>"Developing World" OR	

"Least Developed world" OR<br>"Less Developed world" OR<br>"Lesser Developed world" OR<br>"Under Developed world" OR<br>"Underdeveloped world" OR<br>"Underserved world" OR<br>"deprived world" OR<br>"poor world" OR<br>"poorer world" OR<br>"emerging world" OR<br>"lmic world" OR<br>"lmics world" OR<br>"lami world" OR<br>"transitional world" OR<br>"Developing economy" OR<br>"Least Developed economy" OR<br>"Less Developed economy" OR<br>"Lesser Developed economy" OR<br>"Under Developed economy" OR<br>"Underdeveloped economy" OR<br>"Underserved economy" OR<br>"deprived economy" OR<br>"poor economy" OR<br>"poorer economy" OR<br>"emerging economy" OR<br>"lmic economy" OR<br>"lmics economy" OR<br>"lami economy" OR<br>"transitional economy" OR<br>"Developing economies" OR<br>"Least Developed economies" OR<br>"Less Developed economies" OR<br>"Lesser Developed economies" OR<br>"Under Developed economies" OR<br>"Underdeveloped economies" OR<br>"Underserved economies" OR<br>"deprived economies" OR<br>"poor economies" OR<br>"poorer economies" OR<br>"emerging economies" OR<br>"lmic economies" OR<br>"lmics economies" OR<br>"lami economies" OR<br>"transitional economies" OR<br>"low gross domestic" OR<br>"low gross national" OR<br>"lower gross domestic" OR<br>"lower gross national" OR<br>"global south" OR<br>"third World" OR<br>"LMIC*" OR<br>"low and middle income" OR<br>"low income" OR<br>"middle income" OR<br>"Subsahara*" OR<br>"Central Africa*" OR<br>"Cameroon" OR<br>"Ubangi Shari*" OR<br>"Chad" OR<br>"Congo" OR	

"Zaire" OR<br>"Katanga" OR<br>"Equatorial Guinea*" OR<br>"spanish Guinea*" OR<br>"Gabon*" OR<br>"Sao Tome*" OR<br>"East Africa*" OR<br>"Eastern Africa*" OR<br>"Burundi" OR<br>"Djibouti" OR<br>"Eritrea" OR<br>"Ethiopia" OR<br>"Kenya" OR<br>"Rwanda" OR<br>"Somalia" OR<br>"Sudan" OR<br>"Tanzania" OR<br>"Uganda" OR<br>"Southern africa*" OR<br>"South africa*" OR<br>"Angola" OR<br>"Botswana*" OR<br>"Eswatini*" OR<br>"Lesotho*" OR<br>"Basutoland" OR<br>"Malawi*" OR<br>"Mozambique*" OR<br>"Namibia*" OR<br>"Zambia*" OR<br>"Zimbabwe*" OR<br>"western africa*" OR<br>"west africa*" OR<br>"Benin*" OR<br>"Burkina Faso" OR<br>"upper volta" OR<br>"burkina fasso" OR<br>"Cabo Verde" OR<br>"cape verde" OR<br>"Cote dIvoire" OR<br>"cote d’ ivoire" OR<br>"cote divoire" OR<br>"cote d ivoire" OR<br>"ivory coast" OR<br>"Gambia*" OR<br>"Ghana*" OR<br>"Guinea*" OR<br>"Liberia*" OR<br>"Mali*" OR<br>"Mauritania*" OR<br>"Niger*" OR<br>"Senegal*" OR<br>"Sierra Leone*" OR<br>"Togo*" OR<br>"north africa*" OR<br>"northern africa*" OR<br>"magreb" OR<br>"maghrib" OR<br>"sahara" OR<br>"Algeria" OR<br>"Egypt*" OR	

"United Arab Republic*" OR<br>"Libya*" OR<br>"Morocco*" OR<br>"Ifni*" OR<br>"Tunisia" OR<br>"Djibouti" OR<br>"West Bank*" OR<br>"Gaza strip*" OR<br>"Central asia*" OR<br>"Kazakhstan*" OR<br>"Kyrgyz*" OR<br>"Kirghiz*" OR<br>"Tajikistan" OR<br>"tadjikistan" OR<br>"tadzhik*" OR<br>"Turkmenistan" OR<br>"Uzbekistan" OR<br>"Armenia" OR<br>"Azerbaijan" OR<br>"Georgia" OR<br>"Belarus" OR<br>"Russia" OR<br>"South Asia*" OR<br>"Southern Asia*" OR<br>"Borneo*" OR<br>"Brunei*" OR<br>"Cambodia*" OR<br>"Khmer Republic*" OR<br>"Kampuchea*" OR<br>"Indochina*" OR<br>"Indonesia*" OR<br>"Sabah" OR<br>"Sarawak" OR<br>"Laos*" OR<br>"lao pdr" OR<br>"lao peoples democratic republic" OR<br>"Malaysia*" OR<br>"Malaya" OR<br>"Mekong*" OR<br>"Myanma*" OR<br>"Philippine*" OR<br>"Philipine*" OR<br>"Singapore*" OR<br>"Thailand*" OR<br>"Siam*" OR<br>"Burma*" OR<br>"Timor Leste*" OR<br>"East Timor*" OR<br>"Vietnam*" OR<br>"Bangladesh*" OR<br>"Bhutan*" OR<br>"Maldives*" OR<br>"west Asia*" OR<br>"western Asia*" OR<br>"Afghanistan*" OR<br>"Bahrain*" OR<br>"Iran*" OR<br>"Iraq*" OR<br>"Israel*" OR<br>"Jordan*" OR	

"Kuwait*" OR<br>"Lebanon*" OR<br>"Oman*" OR<br>"Qatar*" OR<br>"Saudi Arabia*" OR<br>"Syria*" OR<br>"Turkey*" OR<br>"Arab Emirate*" OR<br>"Yemen*" OR<br>"Nepal*" OR<br>"Pakistan*" OR<br>"Sri Lanka*" OR<br>"Ceylon*" OR<br>"India*" OR<br>"Andhra Pradesh*" OR<br>"Arunachal Pradesh*" OR<br>"Assam*" OR<br>"Sikkim*" OR<br>"Bihar*" OR<br>"Chhattisgarh*" OR<br>"Goa" OR<br>"Gujarat*" OR<br>"Haryana*" OR<br>"Himachal Pradesh*" OR<br>"Jammu and Kashmir*" OR<br>"Jharkhand*" OR<br>"Kerala*" OR<br>"Karnataka*" OR<br>"Madhya Pradesh*" OR<br>"Maharashtra*" OR<br>"Manipur*" OR<br>"Meghalaya*" OR<br>"Mizoram*" OR<br>"Nagaland*" OR<br>"Odisha*" OR<br>"Punjab*" OR<br>"Rajasthan*" OR<br>"Tamil Nadu*" OR<br>"Telangana*" OR<br>"Tripura*" OR<br>"Uttar Pradesh*" OR<br>"Uttarakhand*" OR<br>"West Bengal*" OR<br>"east Asia*" OR<br>"eastern Asia*" OR<br>"China*" OR<br>"Beijing*" OR<br>"Hong Kong*" OR<br>"Macau*" OR<br>"Tibet*" OR<br>"Korea*" OR<br>"Mongolia*" OR<br>"Fiji*" OR<br>"Micronesia*" OR<br>"Kiribati*" OR<br>"Lao PDR*" OR<br>"Marshall Island*" OR<br>"Papua New Guinea*" OR<br>"Solomon Island" OR<br>"solomon islands" OR	

"melanesia" OR<br>"norfolk island" OR<br>"Tonga*" OR<br>"Tuvalu*" OR<br>"Vanuatu*" OR<br>"Samoa" OR<br>"Albania" OR<br>"Bosnia*" OR<br>"Herzegovina*" OR<br>"Moldova*" OR<br>"North Macedonia*" OR<br>"Northern Macedonia*" OR<br>"Montenegro*" OR<br>"Romania" OR<br>"Bulgaria" OR<br>"Serbia" OR<br>"Ukraine" OR<br>"Kosovo*" OR<br>"Latin America*" OR<br>"Caribbean*" OR<br>"Argentina*" OR<br>"Belize*" OR<br>"Bolivia*" OR<br>"Brazil*" OR<br>"Colombia*" OR<br>"Costa Rica*" OR<br>"Cuba*" OR<br>"Dominica*" OR<br>"Ecuador*" OR<br>"Grenada*" OR<br>"Guatemala*" OR<br>"Guyana*" OR<br>"Honduras*" OR<br>"Haiti*" OR<br>"Jamaica*" OR<br>"St. Lucia*" OR<br>"St Lucia*" OR<br>"Saint Lucia*" OR<br>"Mexico*" OR<br>"Nicaragua*" OR<br>"Panama*" OR<br>"Peru*" OR<br>"Paraguay*" OR<br>"El Salvador*" OR<br>"Suriname*" OR<br>"Grenadines" OR<br>"saint vincent" OR<br>"st vincent" OR<br>"Venezuela" OR<br>"Antigua" OR<br>"Barbuda" OR<br>"Bahama*" OR<br>"Barbados" OR<br>"Guadeloupe" OR<br>"Martinique" OR<br>"Puerto Rico" OR<br>"Saint Kitts*" OR<br>"St Kitts*" OR<br>"Trinidad" OR<br>"Tobago" OR	

"Virgin Island*" OR<br>"Aruba" OR<br>"Curacao" OR<br>"Sint Maarten" OR<br>"West Indies"	

Search strings

P AND I AND X: 3581

Filter 1985 not necessary (Database from 1987)

EconLit

P

("Gender*" OR<br>"genital" OR<br>"Sex" OR<br>"Sexism*" OR<br>"Woman*" OR<br>"Women*" OR<br>"Female*" OR<br>"Girl*")<br>AND<br>("Discrimination*" OR<br>"Bias*" OR<br>"Stigma*")	4882

I

"health*" OR<br>"medical*" OR<br>"diseases" OR<br>"disorder*" OR<br>"illness*" OR<br>"mortalit*" OR<br>"death*" OR<br>"fatalit*" OR<br>"morbidit*" OR<br>"maternal*" OR<br>"maternit*"	78395

X

"Developing Country" OR<br>"Least Developed Country" OR<br>"Less Developed Country" OR<br>"Lesser Developed Country" OR<br>"Under Developed Country" OR<br>"Underdeveloped Country" OR<br>"Underserved country" OR<br>"deprived country" OR<br>"poor country" OR<br>"poorer country" OR<br>"emerging country" OR<br>"lmic country" OR<br>"lmics country" OR<br>"lami country" OR<br>"transitional country" OR<br>"Developing Countries" OR<br>"Least Developed Countries" OR<br>"Less Developed Countries" OR<br>"Lesser Developed Countries" OR<br>"Under Developed Countries" OR<br>"Underdeveloped Countries" OR<br>"Underserved Countries" OR<br>"deprived countries" OR<br>"poor countries" OR<br>"poorer countries" OR<br>"emerging countries" OR<br>"lmic countries" OR<br>"lmics countries" OR<br>"lami countries" OR<br>"transitional countries" OR<br>"Developing Nation" OR	321641

"Least Developed nation" OR<br>"Less Developed nation" OR<br>"Lesser Developed nation" OR<br>"Under Developed nation" OR<br>"Underdeveloped nation" OR<br>"Underserved nation" OR<br>"deprived nation" OR<br>"poor nation" OR<br>"poorer nation" OR<br>"emerging nation" OR<br>"lmic nation" OR<br>"lmics nation" OR<br>"lami nation" OR<br>"transitional nation" OR<br>"Developing Nations" OR<br>"Least Developed nations" OR<br>"Less Developed nations" OR<br>"Lesser Developed nations" OR<br>"Under Developed nations" OR<br>"Underdeveloped nations" OR<br>"Underserved nations" OR<br>"deprived nations" OR<br>"poor nations" OR<br>"poorer nations" OR<br>"emerging nations" OR<br>"lmic nations" OR<br>"lmics nations" OR<br>"lami nations" OR<br>"transitional nations" OR<br>"Developing Population" OR<br>"Least Developed population" OR<br>"Less Developed population" OR<br>"Lesser Developed population" OR<br>"Under Developed population" OR<br>"Underdeveloped population" OR<br>"Underserved population" OR<br>"deprived population" OR<br>"poor population" OR<br>"poorer population" OR<br>"emerging population" OR<br>"lmic population" OR<br>"lmics population" OR<br>"lami population" OR<br>"transitional population" OR<br>"Developing Populations" OR<br>"Least Developed populations" OR<br>"Less Developed populations" OR<br>"Lesser Developed populations" OR<br>"Under Developed populations" OR<br>"Underdeveloped populations" OR<br>"Underserved populations" OR<br>"deprived populations" OR<br>"poor populations" OR<br>"poorer populations" OR<br>"emerging populations" OR<br>"lmic populations" OR<br>"lmics populations" OR<br>"lami populations" OR<br>"transitional populations" OR<br>"Developing World" OR	

"Least Developed world" OR<br>"Less Developed world" OR<br>"Lesser Developed world" OR<br>"Under Developed world" OR<br>"Underdeveloped world" OR<br>"Underserved world" OR<br>"deprived world" OR<br>"poor world" OR<br>"poorer world" OR<br>"emerging world" OR<br>"lmic world" OR<br>"lmics world" OR<br>"lami world" OR<br>"transitional world" OR<br>"Developing economy" OR<br>"Least Developed economy" OR<br>"Less Developed economy" OR<br>"Lesser Developed economy" OR<br>"Under Developed economy" OR<br>"Underdeveloped economy" OR<br>"Underserved economy" OR<br>"deprived economy" OR<br>"poor economy" OR<br>"poorer economy" OR<br>"emerging economy" OR<br>"lmic economy" OR<br>"lmics economy" OR<br>"lami economy" OR<br>"transitional economy" OR<br>"Developing economies" OR<br>"Least Developed economies" OR<br>"Less Developed economies" OR<br>"Lesser Developed economies" OR<br>"Under Developed economies" OR<br>"Underdeveloped economies" OR<br>"Underserved economies" OR<br>"deprived economies" OR<br>"poor economies" OR<br>"poorer economies" OR<br>"emerging economies" OR<br>"lmic economies" OR<br>"lmics economies" OR<br>"lami economies" OR<br>"transitional economies" OR<br>"low gross domestic" OR<br>"low gross national" OR<br>"lower gross domestic" OR<br>"lower gross national" OR<br>"global south" OR<br>"third World" OR<br>"LMIC*" OR<br>"low and middle income" OR<br>"low income" OR<br>"middle income" OR<br>"Subsahara*" OR<br>"Central Africa*" OR<br>"Cameroon" OR<br>"Ubangi Shari*" OR<br>"Chad" OR<br>"Congo" OR	

"Zaire" OR<br>"Katanga" OR<br>"Equatorial Guinea*" OR<br>"spanish Guinea*" OR<br>"Gabon*" OR<br>"Sao Tome*" OR<br>"East Africa*" OR<br>"Eastern Africa*" OR<br>"Burundi" OR<br>"Djibouti" OR<br>"Eritrea" OR<br>"Ethiopia" OR<br>"Kenya" OR<br>"Rwanda" OR<br>"Somalia" OR<br>"Sudan" OR<br>"Tanzania" OR<br>"Uganda" OR<br>"Southern africa*" OR<br>"South africa*" OR<br>"Angola" OR<br>"Botswana*" OR<br>"Eswatini*" OR<br>"Lesotho*" OR<br>"Basutoland" OR<br>"Malawi*" OR<br>"Mozambique*" OR<br>"Namibia*" OR<br>"Zambia*" OR<br>"Zimbabwe*" OR<br>"western africa*" OR<br>"west africa*" OR<br>"Benin*" OR<br>"Burkina Faso" OR<br>"upper volta" OR<br>"burkina fasso" OR<br>"Cabo Verde" OR<br>"cape verde" OR<br>"Cote dIvoire" OR<br>"cote d’ ivoire" OR<br>"cote divoire" OR<br>"cote d ivoire" OR<br>"ivory coast" OR<br>"Gambia*" OR<br>"Ghana*" OR<br>"Guinea*" OR<br>"Liberia*" OR<br>"Mali*" OR<br>"Mauritania*" OR<br>"Niger*" OR<br>"Senegal*" OR<br>"Sierra Leone*" OR<br>"Togo*" OR<br>"north africa*" OR<br>"northern africa*" OR<br>"magreb" OR<br>"maghrib" OR<br>"sahara" OR<br>"Algeria" OR<br>"Egypt*" OR	

"United Arab Republic*" OR<br>"Libya*" OR<br>"Morocco*" OR<br>"Ifni*" OR<br>"Tunisia" OR<br>"Djibouti" OR<br>"West Bank*" OR<br>"Gaza strip*" OR<br>"Central asia*" OR<br>"Kazakhstan*" OR<br>"Kyrgyz*" OR<br>"Kirghiz*" OR<br>"Tajikistan" OR<br>"tadjikistan" OR<br>"tadzhik*" OR<br>"Turkmenistan" OR<br>"Uzbekistan" OR<br>"Armenia" OR<br>"Azerbaijan" OR<br>"Georgia" OR<br>"Belarus" OR<br>"Russia" OR<br>"South Asia*" OR<br>"Southern Asia*" OR<br>"Borneo*" OR<br>"Brunei*" OR<br>"Cambodia*" OR<br>"Khmer Republic*" OR<br>"Kampuchea*" OR<br>"Indochina*" OR<br>"Indonesia*" OR<br>"Sabah" OR<br>"Sarawak" OR<br>"Laos*" OR<br>"lao pdr" OR<br>"lao peoples democratic republic" OR<br>"Malaysia*" OR<br>"Malaya" OR<br>"Mekong*" OR<br>"Myanma*" OR<br>"Philippine*" OR<br>"Philipine*" OR<br>"Singapore*" OR<br>"Thailand*" OR<br>"Siam*" OR<br>"Burma*" OR<br>"Timor Leste*" OR<br>"East Timor*" OR<br>"Vietnam*" OR<br>"Bangladesh*" OR<br>"Bhutan*" OR<br>"Maldives*" OR<br>"west Asia*" OR<br>"western Asia*" OR<br>"Afghanistan*" OR<br>"Bahrain*" OR<br>"Iran*" OR<br>"Iraq*" OR<br>"Israel*" OR<br>"Jordan*" OR	

"Kuwait*" OR<br>"Lebanon*" OR<br>"Oman*" OR<br>"Qatar*" OR<br>"Saudi Arabia*" OR<br>"Syria*" OR<br>"Turkey*" OR<br>"Arab Emirate*" OR<br>"Yemen*" OR<br>"Nepal*" OR<br>"Pakistan*" OR<br>"Sri Lanka*" OR<br>"Ceylon*" OR<br>"India*" OR<br>"Andhra Pradesh*" OR<br>"Arunachal Pradesh*" OR<br>"Assam*" OR<br>"Sikkim*" OR<br>"Bihar*" OR<br>"Chhattisgarh*" OR<br>"Goa" OR<br>"Gujarat*" OR<br>"Haryana*" OR<br>"Himachal Pradesh*" OR<br>"Jammu and Kashmir*" OR<br>"Jharkhand*" OR<br>"Kerala*" OR<br>"Karnataka*" OR<br>"Madhya Pradesh*" OR<br>"Maharashtra*" OR<br>"Manipur*" OR<br>"Meghalaya*" OR<br>"Mizoram*" OR<br>"Nagaland*" OR<br>"Odisha*" OR<br>"Punjab*" OR<br>"Rajasthan*" OR<br>"Tamil Nadu*" OR<br>"Telangana*" OR<br>"Tripura*" OR<br>"Uttar Pradesh*" OR<br>"Uttarakhand*" OR<br>"West Bengal*" OR<br>"east Asia*" OR<br>"eastern Asia*" OR<br>"China*" OR<br>"Beijing*" OR<br>"Hong Kong*" OR<br>"Macau*" OR<br>"Tibet*" OR<br>"Korea*" OR<br>"Mongolia*" OR<br>"Fiji*" OR<br>"Micronesia*" OR<br>"Kiribati*" OR<br>"Lao PDR*" OR<br>"Marshall Island*" OR<br>"Papua New Guinea*" OR<br>"Solomon Island" OR<br>"solomon islands" OR	

"melanesia" OR<br>"norfolk island" OR<br>"Tonga*" OR<br>"Tuvalu*" OR<br>"Vanuatu*" OR<br>"Samoa" OR<br>"Albania" OR<br>"Bosnia*" OR<br>"Herzegovina*" OR<br>"Moldova*" OR<br>"North Macedonia*" OR<br>"Northern Macedonia*" OR<br>"Montenegro*" OR<br>"Romania" OR<br>"Bulgaria" OR<br>"Serbia" OR<br>"Ukraine" OR<br>"Kosovo*" OR<br>"Latin America*" OR<br>"Caribbean*" OR<br>"Argentina*" OR<br>"Belize*" OR<br>"Bolivia*" OR<br>"Brazil*" OR<br>"Colombia*" OR<br>"Costa Rica*" OR<br>"Cuba*" OR<br>"Dominica*" OR<br>"Ecuador*" OR<br>"Grenada*" OR<br>"Guatemala*" OR<br>"Guyana*" OR<br>"Honduras*" OR<br>"Haiti*" OR<br>"Jamaica*" OR<br>"St. Lucia*" OR<br>"St Lucia*" OR<br>"Saint Lucia*" OR<br>"Mexico*" OR<br>"Nicaragua*" OR<br>"Panama*" OR<br>"Peru*" OR<br>"Paraguay*" OR<br>"El Salvador*" OR<br>"Suriname*" OR<br>"Grenadines" OR<br>"saint vincent" OR<br>"st vincent" OR<br>"Venezuela" OR<br>"Antigua" OR<br>"Barbuda" OR<br>"Bahama*" OR<br>"Barbados" OR<br>"Guadeloupe" OR<br>"Martinique" OR<br>"Puerto Rico" OR<br>"Saint Kitts*" OR<br>"St Kitts*" OR<br>"Trinidad" OR<br>"Tobago" OR	

"Virgin Island*" OR<br>"Aruba" OR<br>"Curacao" OR<br>"Sint Maarten" OR<br>"West Indies"	

Search strings

P AND I AND X: 358

Filter from 1985 not applied because Database from 1981 (only one record difference)

ANNEX 2 INCLUDED ARTICLES

Note for reading:

Column id is a unique numbered identifier we attributed to each study that was full-text screened. Here we present only articles that were finally included in the review. This is the same in Annex 2 and Annex 3.

Column “theme” refers to the GBD theme tackled by the concerned study.

Here are the themes numbers

1. Healthcare behaviour
2. Health state
3. Son preference at birth
4. Self-declared discrimination
5. Cultural and legal practices
6. Gender roles
7. Access to economic resources
8. Decision-making
9. Education
10. Violence against women
11. Disrespect and abuse during childbirth

Id	Article	Country	type	Theme
8	Abuya, T., Warren, C. E., Miller, N., Njuki, R., Ndwiga, C., Maranga, A., Mbehero, F., Njeru, A. and Bellows, B.,2015	Kenya	Quantitative	11
11	Afulani, P. A., Kelly, A. M., Buback, L., Asunka, J., Kirumbi, L. and Lyndon, A.,2020	Kenya	Mixed	11
12	Agnihotri, Satish, Palmer-Jones, Richard and Parikh, Ashok,2002	India	Quantitative	3
15	Alkema, L., Chao, F., You, D., Pedersen, J. and Sawyer, C. C.,2014	195 countries	Quantitative	2
16	Altindag, O.,2016	Turkey	Quantitative	2, 3
18	Ananthakrishnan, S. and Nalini, P.,2002	India	Mixed	1, 2, 3
21	Arokiasamy, P.,2004	India	Quantitative	1, 2
22	Asadullah, M. N., Mansoor, N., Randazzo, T. and Wahhaj, Z.,2021	Bengladesh	Quantitative	3

23	Asfaw, A., Lamanna, F. and Klasen, S.,2010	India	Quantitative	1
24	Attané, I.,2009	China	Quantitative	2, 3
25	Ayanore, M. A., Pavlova, M., Biesma, R. and Groot, W.,2017	Ghana	Qualitative	11
26	Azad, A. D., Charles, A. G., Ding, Q., Trickey, A. W. and Wren, S. M.,2020	Malawi	Quantitative	1, 8
31	Bandyopadhyay, M.,2003	India	Qualitative	3
33	Basu, B.,2021	Iran	Quantitative	1
36	Bhalotra, S., Brule, R. and Roy, S.,2020	India	Quantitative	3
37	Bhalotra, S., Chakravarty, A., Mookherjee, D. and Pino, F. J.,2019	India	Quantitative	2
39	Bharadwaj, P. and Lakdawala, L. K.,2013	India, Bengladesh, China, Pakistan, Ghana, Sri Lanka, Thailand	Quantitative	1, 2
41	Bhat, P. N. and Zavier, A. J.,2003	India	Quantitative	3
42	Bishop, J. A., Liu, H. Y. and Zeager, L. A.,2011	China	Quantitative	1
44	Boccolini, C. S., Boccolini Pde, M., Damacena, G. N., Ferreira, A. P. and Szwarcwald, C. L.,2016	Brazil	Quantitative	4
45	Bohren, M. A., Mehrtash, H., Fawole, B., Maung, T. M., Balde, M. D., Maya, E., Thwin, S. S., Aderoba, A. K., Vogel, J. P., Irinyenikan, T. A., Adeyanju, A. O., Mon, N. O., Adu-Bonsaffoh, K., Landoulsi, S., Guure, C., Adanu, R., Diallo, B. A., Gülmezoglu, A. M., Soumah, A. M., Sall, A. O. and Tunçalp, Ö,2019	Nigeria, Ghana, Guinea, and Myanmar	Quantitative	11
46	Bui, H. T. T., Le, T. M., Van Pham, T., Doan, D. T. T., Nguyen, D. A., Nguyen, C. C. and Duong, D. M.,2018	Vietnam	Quantitative	6, 7, 8, 10
48	Cai, Z., Canetto, S. S., Chang, Q. and Yip, P. S. F.,2021	176 countries	Quantitative	5, 7, 8, 10
49	Çalıkoglu, E. O., Aras, A., Hamza, M., Aydin, A., Nacakgedigi, O. and Koga, P. M.,2018	Turkey	Quantitative	6, 7, 8, 9, 10
50	Calu Costa, J., Wehrmeister, F. C., Barros, A. J. and Victora, C. G.,2017	57 LMICs	Quantitative	1
53	Chakravarty, A.,2015	Egypt	Quantitative	1
56	Chaudhuri, S.,2015	India	Quantitative	1
58	Choi, J. Y. and Lee, S. H.,2006	India	Quantitative	1
63	Costa, J. C., Weber, A. M., Abdalla, S., Darmstadt, G. L. and Victora, C. G.,2021	80 LMICs	Quantitative	2
66	Dancer, D., Rammohan, A. and Smith, M. D.,2008	Bengladesh	Quantitative	2
68	Dasgupta, M. and Bhat, P. N. M.,1997	India, China, South Korea	Quantitative	3

70	Dessalegn, M., Ayele, M., Hailu, Y., Addisu, G., Abebe, S., Solomon, H., Mogess, G. and Stulz, V.,2020	Ethiopia	Qualitative	6
71	Diamond-Smith, N., Treleaven, E., Murthy, N. and Sudhinaraset, M.,2017	India	Quantitative	11
73	Doyle, K., Kazimbaya, S., Levtov, R., Banerjee, J., Betron, M., Sethi, R., Kayirangwa, M. R., Vlahovicova, K., Sayinzoga, F. and Morgan, R.,2021	Rwanda	Mixed	11
74	Dubuc, S. and Sivia, D. S.,2018	India	Quantitative	3
77	Ene-Obong, H. N., Enugu, G. I. and Uwaegbute, A. C.,2001	Nigeria	Mixed	1, 2
80	Frongillo, E. A. and Begin, F.,1993	Guatemala	Quantitative	1, 2
81	Ganatra, B. and Hirve, S.,1994	India	Quantitative	1
82	Gangadharan, Lata and Maitra, Pushkar,2000	Pakistan	Quantitative	2
83	Gellatly, C. and Petrie, M.,2017	India	Quantitative	2, 3
88	Grabowski, R. and Self, S.,2013	India	Quantitative	1
89	Guilmoto, C. Z.,2015	Indonesia	Quantitative	3
90	Guilmoto, C. Z., Saikia, N., Tamrakar, V. and Bora, J. K.,2018	India	Quantitative	2
91	Hadley, C., Lindstrom, D., Tessema, F. and Belachew, T.,2008	Ethiopia	Quantitative	1, 2
92	Hafeez, Naima and Quintana-Domeque, Climent,2018	Pakistan	Quantitative	1
94	Hathi, P., Coffey, D., Thorat, A. and Khalid, N.,2021	India	Quantitative	5, 8
95	Hazarika, G.,2000	Pakistan	Quantitative	1
97	Hinton, R. and Earnest, J.,2010	Papua New Guinea	Qualitative	1, 2
99	Ijadunola, M. Y., Olotu, E. A., Oyedun, O. O., Eferakeya, S. O., Ilesanmi, F. I., Fagbemi, A. T. and Fasae, O. C.,2019	Nigeria	Quantitative	11
100	Iyer, A., Sen, G. and George, A.,2007	India	Quantitative	1
102	Jain, T.,2014	India	Quantitative	3
103	Jamal, Haroon,2018	Pakistan	Quantitative	7, 8, 9, 10
105	Kabeer, N., Huq, L. and Mahmud, S.,2014	Bangladesh	Mixed	3
107	Kapoor, M., Agrawal, D., Ravi, S., Roy, A., Subramanian, S. V. and Guleria, R.,2019	India	Quantitative	1
108	Karbeyaz, K., Yeti Ş, Y., Güneş, A. and Şi Mşek, Ü,2018	turkey	Quantitative	10
110	Kashyap, R. and Behrman, J.,2020	India	Quantitative	2
112	Kaur, R. and Garg, S.,2010	India	Qualitative	10

115	Kira, I. A., Shuwiekh, H., Kucharska, J., Abu-Ras, W. and Bujold-Bugeaud, M.,2020	Poland and Egypt	Quantitative	5, 8
116	Klasen, S.,1994	India, China, Egypt, Bangladesh, Pakistan, Nepal	Quantitative	2
120	Kosterina, E., Horne, S. G. and Lamb, S.,2021	Kyrgyzstan	Quantitative	6, 10
121	Kumar, K., Singh, A., James, K. S., Mcdougal, L. and Raj, A.,2020	India	Quantitative	1
127	Leone, T., Matthews, Z. and Dalla Zuanna, G.,2003	Nepal	Quantitative	1, 3
128	Li, S., Zhu, C. and Feldman, M. W.,2004	China	Quantitative	1, 2
129	Lodenstein, E.; Pedersen, K.; Botha, K.; Broerse, J. E. W.; Dieleman, M.,2018	Malawi	Qualitative	11
130	Lofstedt, P., Luo, S. S. and Johansson, A.,2004	China	Quantitative	3
131	Madan, Sonu and Khanna, Vandana,2011	India	Quantitative	2, 3
133	Maitra, P. and Rammohan, A.,2011	India	Quantitative	2
135	Masud, Faham and Farooq, Shujaat,2012	Pakistan	Quantitative	1
136	Mayra, K., Matthews, Z. and Padmadas, S. S.,2021	India	Qualitative	11
142	Mihret, H., Atnafu, A., Gebremedhin, T. and Dellie, E.,2020	Ethiopia	Mixed	11
143	Milazzo, A.,2018	India	Quantitative	1, 2
148	Mishra, V., Roy, T. K. and Retherford, R. D.,2004	India	Quantitative	1, 2
149	Moestue, H.,2009	Bangladesh	Quantitative	2
150	Mohanty, S. K. and Rajbhar, M.,2014	India	Quantitative	2, 3
151	Molla, M., Muleta, M., Betemariam, W., Fesseha, N. and Karim, A.,2017	Eithiopia	Qualitative	11
152	Mondal, B. and Dubey, J. D.,2020	India	Quantitative	1
155	Morduch, J. J. and Stern, H. S.,1997	Bengladesh	Quantitative	2
157	Morgan, R., Tetui, M., Muhumuza Kananura, R., Ekirapa-Kiracho, E. and George, A. S.,2017	Uganda	Qualitative	6
158	Muhuri, P. K. and Preston, S. H.,1991	Bangladesh	Quantitative	2
160	Murthi, M., Guio, A. C. and Dreze, J.,1995	India	Quantitative	2, 3
162	Nawab, T., Erum, U., Amir, A., Khalique, N., Ansari, M. A. and Chauhan, A.,2019	India	Quantitative	11
164	Obermeyer, C. M. and Cárdenas, R.,1997	Morocco and Tunisia	Quantitative	2
165	Oduenyi, C., Banerjee, J., Adetiloye, O., Rawlins, B., Okoli, U., Orji, B., Ugwa, E., Ishola, G. and Betron, M.,2021	Nigeria	Quantitative	11

167	Olofinbiyi, B. A., Awoleke, J. O., Atiba, B. P., Olaogun, O. D., Olofinbiyi, R. O. and Awoleke, A. O.,2021	Nigeria	Quantitative	3
168	Oluoch-Aridi, J., Smith-Oka, V., Milan, E. and Dowd, R.,2018	Kenya	Qualitative	11
172	Patra, Nilanjan,2011	India	Quantitative	2
173	Patra, Nilanjan,2011	India	Quantitative	1
178	Rahman, M., Nakamura, K., Seino, K. and Kizuki, M.,2013	Bangladesh	Quantitative	8, 10
179	Raj, A., Dey, A., Boyce, S., Seth, A., Bora, S., Chandurkar, D., Hay, K., Singh, K., Das, A. K., Chakraverty, A., Ramakrishnan, A., Shetye, M., Saggurti, N. and Silverman, J. G.,2017	India	Quantitative	11
180	Rajan, S. and Morgan, S. P.,2018	India	Quantitative	1
182	Rosenstock, S., Katz, J., Mullany, L. C., Khatry, S. K., Leclerq, S. C., Darmstadt, G. L. and Tielsch, J. M.,2015	Nepal	Quantitative	1, 2
184	Roy, T. K. and Chattopadhyay, A.,2012	India	Quantitative	3
191	Sheferaw, E. D., Mengesha, T. Z. and Wase, S. B. 2016	Ethiopia	Quantitative	11
192	Singh, A.,2012	India	Quantitative	1
193	Singh, Abhishek and Patel, Sangram Kishor,2017	India	Quantitative	1, 2
194	Sinha, A., Mcroy, R. G., Berkman, B. and Sutherland, M.,2017	India	Quantitative	1
196	Srivastava, S. P. and Nayak, N. P.,1995	India	Quantitative	1
198	Sudha, S. and Rajan, S. I.,1999	India	Quantitative	3
201	Treleaven, E., Toan, P. N., Le, D. N., Diamond-Smith, N., Partridge, J. C. and Le, H. T.,2016	Vietnam	Quantitative	1
202	Ukke, G. G., Gurara, M. K. and Boynito, W. G.,2019	Ethiopia	Quantitative	11
208	Warren, C. E., Njue, R., Ndwiga, C. and Abuya, T.,2017	Kenya	Qualitative	11
210	Weitzman, A.,2020	India	Quantitative	10
214	Organisation for Economic, Co-operation; Development,2010	all no OECD countries	Quantitative	7, 10
215	T. Herath, D. Guruge, M. Fernando, S. Jayarathna and L. Senarathna,2018	Sri Lanka	Mixed	6
216	Jayachandran, S and Kuziemko, I, 2011	India	Quantitative	1, 3
217	Pulerwitz, J and Barker,G, 2008	Brazil	Quantitative	6, 10
220	Kira, I, Hanaa, S and Bujold-Bugeaud M,2015	Egypt	Quantitative	6

228	Abramsky T., Harvey, S., Mosha, N., Mtolela, G., Gibbs, A., Mshana, G., Lees, S., Kapiga, S., Stockl, H2022	Tanzania	quantitative	10
234	Adediran, Olanrewaju Adewole2024	South Africa	quantitative	8
239	Adu-Bonsaffoh, K., Mehrtash, H., Guure, C., Maya, E., Vogel, J. P., Irinyenikan, T. A., Aderoba, A. K., Balde, M. D., Adanu, R., Bohren, M. A., Tuncalp, A2021	Ghana, Guinea and Nigeria	quantitative	11
242	Agarwal Goel, Prarthna and Katewa, Neeraj2024	India	quantitative	8, 10
247	Agu, I. C., Eze, I. I., Agu, C. I., Agu, O., Mbachu, C. O., Onwujekwe, O.2024	Nigeria	quantitative	10
248	Aguero, Jorge M., Frisancho, Veronica2022	Peru	quantitative	10
251	Ahmed, H. and Khalid, H.2023	Pakistan	quantitative	1, 2
252	Ahsan, M. N., Thakur, S.2024	India	quantitative	2
255	Ajefu, J.2024	India	quantitative	7
256	Ajefu, J. B., Uchenna, E., Singh, N., Ali, S. Z.2022	Kenya	quantitative	7
259	Alam, N., Rahman, M. M., Bashar, M., Ahmed, A., Ali, T., Haider, M. M.2022	Bangladesh	quantitative	1, 3
261	Alemu, R., Masters, W. A., Finaret, A. B.2023	72 LMIC	quantitative	2
263	Ali, T. S., Nadeem, S., Memon, Z., Soofi, S., Madhani, F., Karim, Y., Mohammad, S., Bhutta, Z. A.2022	Pakistan	qualitative	3, 6
264	Alinsato, A. S., Alakonon, Calixe B., Bassongui, N.2024	Benin	quantitative	8, 9, 10
271	Ang, C. W., Lai, S. L.2022	Cambodia	quantitative	8, 10
273	Anti, Sebastian and Zhang, Zhihui2023	Cambodia	quantitative	1, 7, 8, 10
274	Anukriti, S.and Bhalotra, S. and Tam, E. H. F2022	India	quantitative	1, 3
281	Bandyopadhyay, S., Sarkar, S., Sensarma, R.2023	India	quantitative	10
284	Bawuah, A., Sarfo, M., Biney, G. K., Appiah, F., Baatiema, L., Yaya, S2025	Nigeria	quantitative	3
285	Becquet, Valentine, Sacco, Nicolas, Pardo, Ignacio2022	Cambodia, Indonesia, Laos, Malaysia, Vietnam, Thailand, Argentina, Brazil, Colombia, Ecuador, Mexico, Uruguay	quantitative	3
290	Bhalotra, Sonia, Clarke, D, Gomes, J F, Venkataramani, A2023	Afghanistan, Algeria, Bangladesh, Burundi, China, Djibouti, Eritrea, Haiti, Iraq, Jordan, Kenya, Morocco, Niger, Pakistan,	quantitative	5

		Rwanda, Saudi Arabia, South Sudan, Sudan, Swaziland, Tanzania, Uganda, and Zimbabwe		
291	Bhatnagar, I.2023	India	quantitative	3
292	Bhattacharjee, Shampa, and Chaudhuri, Arka Roy2024	India	quantitative	2
295	Boggiano, Barbara2024	Paraguay	quantitative	6, 7, 9, 10
298	Bose, Nayana, and Das, Shreyasee2024	India	quantitative	7
300	Bulte, Erwin, Hsieh, Chih-Sheng, Tu, Qin, Wang, Ruixin2021	China	quantitative	3
310	Chao, F., Masquelier, B., You, D., Hug, L., Liu, Y., Sharrow, D., Rue, H., Ombao, H., Alkema, L2023	200 countries	quantitative	2
311	Chao, F.2022	Nepal	quantitative	3
313	Chen, Futing, Wang, Cuntong2024	China	quantitative	9
315	Chen, L.2024	China	quantitative	10
319	Choudhary, S. M., Kubde, S., Ukey, U. U., Agrawal, S. B., Shinde, R. R2022	India	quantitative	1, 2, 3
320	Christopher, E., Drame, N. D., Leyna, G. H., Killewo, J., Barnighausen, T.2022	Tanzania	quantitative	10
322	Clark, C. J., Bergenfeld, I., Cheong, Y. F., Najera, H., Sardinha, L., Garca-Moreno, C., Heise, L.2023	47 countries	quantitative	10
324	Costenbader, E., Memmott, C., Litvin, K., Green, M., Mba-Oduwusi, N., Offiaeli, I., Hajeebhoy, N2025	Nigeria	qualitative	6, 8
327	Dasgupta, A., Sharma, A.2024	India	quantitative	1, 2, 3
328	Dasgupta, A., Sharma, A2024	India	quantitative	1
329	Datt, Gaurav2022	China and India	quantitative	2, 3
331	Dawadi, P., Bhatta, A. S., Rajbanshi, L., Gautam, R2024	Nepal	quantitative	3
335	Diaz, Juan-Jose, Saldarriaga, Victor2023	Peru	quantitative	10
336	Dong, Z., Alhaj-Yaseen, Y., Jiao, Y., Zhong, Y.2021	China	quantitative	3
337	Dupas, P., Jain, R2024	India	quantitative	1
338	Ebert, C., Vollmer, S.2022	India	quantitative	1, 2, 3
339	Echavarri, R.2025	India	quantitative	2, 3
340	Efobi, Uchenna R., Ajefu, Joseph B.2023	Nigeria	quantitative	8

341	Elmira, E. S., Chichaibelu, B. B., Qaim, M.2024	Indonesia	quantitative	1
344	Farnworth, C. R., Jumba, H., Otieno, P.l E., Galie, A., Ouma, E., Flax, V. L.2023	Rwanda	qualitative	6
345	Fei, Z.2025	China	quantitative	3
346	Fenske, J., Gupta, B., Neumann, C.2022	Bangladesh, Burma, India, and Pakistan	quantitative	3
347	Fledderjohann, J., Channon, M2022	Nepal	quantitative	1, 2
348	Garcia, Jorge Luis2024	China	quantitative	3
352	Ghatak, D., Sahoo, S., Sarkar, S., Sharma, V2024	India	quantitative	1
353	Gibson, M. A.2022	Ethiopia	quantitative	10
357	Gu, X., Li, H., Peng, L.2022	China	quantitative	2, 5, 8
358	Guimbeau, A., Ji, Xinde J., Menon, N., Rodgers, Y. van der Meulen2023	India	quantitative	10
359	Gulema, H., Demissie, M., Worku, A., Yadeta, T. A., Tewahido, D., Berhane, Y2024	Ethiopia	qualitative	6
360	Guo, R, Wang, Q, Yi, J, Zhang, J2022	China	quantitative	3
362	Gupta, Rakesh, Goyal, Aarti, Kaur, Gurjinder, Purohit, Neha, Prinja, Shankar2021	India	quantitative	3
372	Hiscox, L. V., Fairchild, G., Donald, K. A., Groenewold, N. A., Koen, N., Roos, A., Narr, K. L., Lawrence, M., Hoffman, N., Wedderburn, C. J., Barnett, W., Zar, H. J., Stein, D. J., Halligan, S. L.2023	South Africa	quantitative	10
373	Ho-Foster, A., Machisa, M. T., Moalusi, L. R., Christofides, N2025	Botswana	quantitative	8, 10
374	Hossain, M., Asadullah, M. Niaz2021	Bangladesh	quantitative	1
377	Ibupoto, M. H., Shah, A. A. Sang, A.2025	Pakistan	quantitative	2
378	Inamdar, V, Tagat, A., Parekh, A.2023	India	quantitative	2, 7, 8, 10
379	Ishaque, M., Hazerjian, J., Brooks, M. I., Sarosh, T., Latif, M., Ali, M 2025	Pakistan	quantitative	1, 3
380	Javadekar, S., Saxena, K.2025	India	quantitative	3
381	Javed, R. and Mughal, M.2022	Pakistan	quantitative	3
385	Kanougiya, S., Sivakami, M, Daruwalla, N., Osrin, D.,2022	India	quantitative	10
387	Kasaye, H., Scarf, V., Sheehy, A., Baird, K.2024	Ethiopia	quantitative	11
388	Kasaye, H., Scarf, V., Sheehy, A., Baird, K.2024	Ethiopia	qualitative	11
392	Kilgallen, J. A., Schaffnit, S. B., Kumogola, Y., Urassa, M., Lawson, D. W.2025	Tanzania	quantitative	10

397	Kumo, K., Perugini, C.2024	Kazakhstan, Kyrgyzstan, Tajikistan, Uzbekistan, Azerbaijan, Kuwait, Qatar, Iran, Iraq, Jordan, Lebanon, and Pakistan	quantitative	5, 7, 9
401	Le, Duc Dung and Giang, Long Thanh2025	Vietnam	quantitative	10
402	Le, K.2022	66 countries	quantitative	2, 3
405	Li, Z., Yang, H., Zhu, X., Xie, L.2021	China	quantitative	6, 10
406	Liu, Huanwen, Dong, Yanfang, Luo, Changfu2024	China	quantitative	3
411	Martínez-Baquero, L. C.2024	Colombia	quantitative	6
413	Maxwell, L., Khan, Z., Yount, K. M.2022	15 countries	quantitative	5
414	Mayra, K., Matthews, Z., Padmadas, S. S.2022	India	qualitative	11
417	Melkam, M., Fente, B. M., Negussie, Y. M., Asmare, Z. A., Asebe, H. A., Seifu, B. L., Bezie, M. M., Asnake, A. A2024	Burundi, Comoros, Ethiopia, Kenya, Madagascar, Malawi, Mozambique, Rwanda, Tanzania, Uganda, Zambia, and Zimbabwe	quantitative	10
420	Mishra, A., Parasnis, J2022	India	quantitative	3
421	Mookerjee, M., Ojha, M., Roy, S.2022	India	quantitative	3
423	Mughal, M., Javed, R., Lorey, T.2023	Pakistan	quantitative	1, 3
428	Nata, Duvvury, Jema, Haji, Dereje, Kifle, Mrinal, Chadha, Caroline, Forde2023	Ethiopia	quantitative	7, 10
429	Nath, Shanjukta2023	India	quantitative	3
430	Naved, R. T., Antu, J. F., Parvin, K., Haider, M. M., Hanifi, S. M. A.2024	Bangladesh	quantitative	3
432	Nguyen, M., Le, K.2022	67 LMICs	quantitative	3
434	Oloniniyi, I. O., Ibigbami, O., Oginni, O. A., Ugo, V., Adelola, A., Esan, O. A., Amiola, A., Daropale, O., Ebuka, M. Esan, O., Mapayi, B2023	Nigeria	quantitative	10
435	Onyango, E. O., Elliott, S. J.2023	Kenya	qualitative	10
436	Osarfo, J., Ampofo, G. D., Tagbor, H. K2025	Ghana	quantitative	1
437	Ouahid, H., Mansouri, A., Sebbani, M., Nouari, N., Khachay, F. E., Cherkaoui, M., Amine, M., Adarmouch, L.2023	Morocco	qualitative	6
438	Ousman, S. K., Gebremariam, M. K., Sundby, J., Magnus, J. H.2022	Ethiopia	quantitative	8, 9, 10
440	Pakrashi, Debayan2024	India	quantitative	10

445	Pesando, Luca Maria2022	Angola, Burundi, Ethiopia, Malawi, Tanzania, Uganda, Zimbabwe, Haiti, Nepal and Timor-Leste	quantitative	10
447	Pörtner, C. C.2022	India	quantitative	3
451	Rastogi, Garima and Sharma, Anisha2022	India	quantitative	3, 9
454	Robitaille, Marie-Claire, Milla, Joniada2024	Albania	quantitative	3
458	Rodriguez, Z.2022	India	quantitative	7, 10
461	Sattar, T., Ahmad, S., Asim, M2022	Pakistan	qualitative	10
462	Schief, Matthias, Vogt, Sonja, Efferson, Charles2021	Armenia	quantitative	3
468	Shayestefar, M., Saffari, M., Gholamhosseinzadeh, R., Nobahar, M., Mirmohammadkhani, M., Shahcheragh, S. H., Khosravi, Z2023	Iran	mixed method	10
470	Shrestha, R.2023	Nepal	quantitative	10
471	Shrestha, Vinish and Jung, Juergen2023	Nepal	quantitative	2
479	Song, Ruixia, Li, Shuzhuo, Eklund, Lisa2022	China	quantitative	3, 6
481	Subedi, S, Katz, J.,Erchick DJ .2022	nepal	quantitative	2, 3
482	Sviatschi, M. M., Trako, I.2024	Peru	quantitative	10
484	Tandel, Vaidehi, Dutta, Arnab, Gandhi, Sahil, Narayanan, Ashwini2023	India	quantitative	7
485	Tang, D., Gao, X., Cai, J., Coyte, P. C 2022	China	quantitative	3
487	Treleaven, E., Pham, TN, Nguyen, AD, Diamond-Smith, N. 2021	Vietnam	quantitative	3
490	Vu, Tien Manh, Yamada, Hiroyuki2024	Vietnam	quantitative	2, 7, 9
491	Wang, Shing-Yi2023	China	quantitative	1, 2, 8
492	Wang, X. Q., Fu, Z. X., Shi, J. L.2025	China	quantitative	3
494	Wiem, B. A., Hela, S., Jihen, J., Hatem, K., Narjes, K., Malek, Z., Fatma, D., Samir, M., Zouhir, H2023	Tunisia	quantitative	10
496	Xiong, Wanru2022	China	quantitative	2, 3
497	Yalley, A. A.2024	Ghana	qualitative	11
498	Yang, Wei, Spencer, Byron G.2022	China	quantitative	3
499	Yao, Yuxin, Zhang, Min, Li, Yixian2023	China	quantitative	3
500	Ye, Y., He, Q., Li, Q., An, L.2024	China	quantitative	2

503	Yount, K. M., Durr, R. L., Bergenfeld, I., Sharma, S., Clark, C. J., Laterra, A., Kalra, S., Sprinkel, A., Cheong, Y. F2023	Nepal	quantitative	6, 8, 10
506	Zaidi, B.2024	Pakistan	quantitative	2
507	Zhao, Menghan, Gao, Weijie, Zhang, Youlang2022	China	quantitative	8, 10
508	Zhao, S., Liu, S., Gao, J., Ma, N., Chen, S., Chandan, J. S., Kim, R., Karoli, P., Niyi, J. L., Rajeev, J., Zemene, M. A., Khan, M. N., Msuya, H. M., Lu, C.2025	49 LMICs	quantitative	10
509	Zhou, Dong, Li, Xue, Su, Yaqin2021	China	quantitative	6, 10

Annex 3: definitions of GBD in the included studies

Indication for reading:

Column id corresponds to the same ids as in Annex 2.

Column “comments” provides additional information on the definitions: “Examples” stands for when the definition is made of examples, “consequences” for when GBD is defined by its consequences, and “specific” for when the definition concerns specific forms of GBD

id	**Definition of GBD**	**Comments**
12	"Discrimination against women operating through unequal access to life sustaining inputs such as food, nutrition and health care"	Examples
24	2 forms explicated " There is a consensus among analysts that this trend primarily reflects two forms of discrimination against females: sex-selective abortion that has a great impact on the sex ratio at birth, and neglect of preventive and curative health care for girls, which leads to excess mortality among females at the youngest ages"	Examples
50	Gender bias is a multidimensional social construct, in which different values are attributed to men and women in a given society, which can lead to preferential treatment of children of a given sex [1,2]; the use of this concept refers to a system of relations including sex, but goes beyond biological differences	
80	Gender bias is noted in social and economic status, occupation, access to education and health services, and food allocation. Gender bias may be attributable to reasons of religious, economic or cultural origin and is more likely to be common in Asian societies and in patriarchal societies. Where gender bias does exist, discriminatory practices favoring male children may have serious implications for the health of girls and may even jeopardize their ability to fulfill their future roles as mothers, workers and members of society (Ravindran 1986).	
94	An important form of gender discrimination in India occurs in the ways in which food is distributed within households. For example, girl babies are breastfed for shorter periods than boy babies [5], girl children are given less and worse food than boy children [6, 7], and women, despite doing almost all of the cooking, are often expected to eat last [8]. When women eat their meals after men, they often eat leftover food that is of lower quality than what they would consume if men and women ate together [9].	Example
100	Gendered practices resulting from biased values and norms may function to limit treatment for women, whether or not the household is able to afford health care. But gender bias may also take the form of rationing health care differently for women and men (girls and boys) in situations of poverty or growing resource constraints. We refer to these two forms of gender bias as pure bias and rationing bias, respectively.	
108	Russell defined the term femicide as the killing of females by males because they are female. Any behavior based on gender discrimination that causes physical, sexual or psychological damage or impairment in women is defined as violence against women.	Specific
110	The dominant demographic explanation for the female mortality disadvantage in India has been that parents invest more resources (e.g., immunizations, medical treatment, nutrition) in sons relative to daughters—a set of processes that we refer to as explicit discrimination—leading to girls’ poorer health status and, consequently, higher mortality (Caldwell and Caldwell 1990; Caldwell et al. 1982; Das Gupta 1987; Miller 1981).	Specific
112	Domestic violence can be described as when one adult in a relationship misuses power to control another. It is the establishment of control and fear in a relationship through violence and other forms of abuse. This violence can take the form of physical assault, psychological abuse, social abuse, financial abuse, or sexual assault.	Specific

113	Norms are perpetuated by social traditions that govern and constrain behaviours of both women and men, and by social institutions that produce laws and codes of conduct that maintain gender inequities. All countries, to some degree, experience tensions between emerging roles for women in society and expressions of their social, economic, and political rights, with traditional kinship concepts of women's roles. Such tensions are magnified in countries regarded as being in development.	
115	Structural and systemic sexism theory put gender discrimination (GD) as one of the social-structural violence against women (Farmer 1996). The developmentally-based trauma framework (DBTF) provides a traumatology perspective on GD (e.g., Kira et al. 2015b). DBTF emphasize that GD has deep traumatic and insidious dynamics that result in adverse mental health outcomes for both genders (e.g., Kira et al. 2017).	
116	Gender discrimination resulting in excess female mortality has attracted considerable attention since the appearance of research showing large survival disadvantages for women and girls in many parts of the developing world (Kynch and Sen, 1983; Kynch, 1985; Sen, 1989; D'Souza and Chen, 1980).' One way to estimate the magnitude of the female disadvantage is to estimate how many additional females would be alive today in the absence of past and present gender discrimination in mortality.	Consequences
120	Ambivalent Sexism (AS) is defined as holding both hostile and benevolent sexist attitudes toward women and AS has been found to correlate with women's greater acceptance of violence against women in the forms of domestic violence and rape myths (Chapleau et al., 2007; Glick et al., 2002; Lila et al., 2013; Koepke et al., 2014; Marques-Fagundes et al., 2015; Yamawaki, 2007), including in international samples (Expósito et al., 2010; Glick et al., 2002; Marques-Fagundes et al., 2015; Yamawaki, Ostenson and Brown, 2009).	
127	Son preference is generally viewed as a socially determined bias: In a patriarchal society, couples prefer to raise a child who has the culturally accepted characteristics, status and economic potential associated with the male gender. This preference often influences behavior and may result in gender biases that negatively affect girls' and women's welfare, health and survival. Thus, preference may lead to discrimination.	Specific
128	This excess is usually explained in terms of discrimination against girls in socioeconomic and health-related behavioural and environmental factors, such as nutrition, food and health care (Hill & Upchurch, 1995). Moreover, excess female child mortality also reflects the low status of females, especially girls, and the related strong preference forsons (Waldron, 1983).	Example
129	Norms express particular values and relations of power, and they are gendered in the following ways:<br>1) in their formulation, they articulate societal expectations regarding the roles, behaviours and attitudes that are considered appropriate for men and women; they may privilege either men's or women's interests, such as in the case of male involvement or gender quota in local elections;<br>2) norms are always subject to interpretation and are developed, accepted, maintained, circumvented, manipulated or contested by actors who are operating according to hierarchical power relationships, based on gender (among others);<br>3) in practice (implementation), they are applied differently to men and women, and different groups of women; and<br>4) they have differential effects for men and women (e.g. health outcomes or gender equality outcomes).	
135	The gender discrimination against girls in health care can be seen in two ways; active elimination and passive elimination. Active elimination can be seen in terms of female infanticide and sex-selective abortions while in the later, the girls are neglected in preventive and curative health care treatment.	
136	Sen et al. defines disrespect and abuse during childbirth as a violation of women's dignity and reflects on it from an intersectional angle, stating that race, ethnicity, economic status, marital status, disability, gender identity and sexual orientation may increase women's vulnerability to mistreatment. Additionally, abusive and sexist comments that health workers make to women at the time of childbirth may be normalised, endured, and tolerated as part of low expectations around care at birth.	Specific

140	We refer to gender as a social construct, i.e. as a socially conditioned subjective identity linked to a socially ascribed gender role and position, which is often associated with a lower social status of women, leading to differential health outcomes for women as compared to men. Gendered health differentials, in contrast to biological differences, originate in the different socially constructed risk factors for men and women and in the gendered barriers to accessing healthcare, which affect women differently than men. As children usually gain access to healthcare through women, gendered barriers affect children as well.	
142	Disrespect and abuse (D&A) during childbirth determine the quality of maternal care and it is one of the indicators of a violation of women's basic human rights. It includes both the behavior of healthcare providers and structural deficiencies from human rights standards of good quality care or recommended professional practices.	Specific
165	"In this study, we define gender dynamics as relation-ships and interactions among girls, boys, women, and men. Gender-sensitivity, in this context, refers to providers' knowledge, attitudes, practices, and beliefs about gender equity that take into account gender differences in access to health information, service delivery, and health outcomes."	
215	Gender Based Violence (GBV) means any act of violence occurring based on gender that results in, or is likely to result in physical, sexual, psychological or economic harm or suffering for women, including threats or such acts, coercion or arbitrary deprivations of liberty, whether occurring in public or private life. The most pervasive form of gender violence is abuse of women by intimate male partners, and this is known as intimate partner violence or domestic violence for this study.	Specific
217	Gender norms: "defined here as social expectations for appropriate behaviors of men as compared to women"	
219	"We view sexism as a multidimensional construct that encompasses two sets of sexist attitudes: hostile and benevolent sexism. Hostile sexism needs little explanation; by it we mean those aspects of sexism that fit Allport's (1954) classic definition of prejudice. We define benevolent sexism as a set of interrelated attitudes toward women that are sexist in terms of viewing women stereotypically and in restricted roles but that are subjectively positive in feeling tone (for the perceiver) and also tend to elicit behaviours typically categorized as prosocial (e.g., helping) or intimacy-seeking (e.g., self-disclosure).	
228	Gender-based violence (GBV) is defined by the United Nations in its Declaration on the Elimination of Violence against Women as an act that results in, or is likely to result in, physical, sexual or mental harm or suffering to women, including threats of such acts, coercion, or arbitrary deprivation of liberty, whether occurring in public or private life	Specific
263	Gender discrimination refers to any situation where a person is treated diferently because they are male or female, rather than based on their competency or proficiency	
271	Violence against women refers to any act that leads to or is likely to lead to physical, sexual, or psychological harm to women, including threats and coercion or arbitrary deprivation of liberty, whether occurring publicly or in private life (United Nations, 1993, 1995)	Specific
373	Nonetheless, endorsement of scale items reflects an acknowledgement of individual and perceived community norms of an important form of gender discrimination. In the absence of a strong predictor of depression, like physical or sexual IPV, gender discrimination may independently impede healthy lifestyles that support mental health.	Example
417	The World Health Organization (WHO) defines intimate partner violence as the deliberate act of an intimate partner or former spouse that results in sexual misconduct, severe physical harm, emotional abuse, or dominating activities [1]. Intimate partner violence is the most prevalent type of violence against women, with major health consequences, and is more likely to occur in homes rather than on street level. Intimate partner violence increases the risk of gynecological, neurological, and stressful problems for women	Specific
500	"Additionally, boy preference, as a form of intrafamily gender discrimination (Song & Burgard, 2008), only manifests within opposite-sex sibling pairs, not same-sex pairs (Guo et al., 2022)."	Specific
